# Differential Proteomic Analysis of Chinese Giant Salamander Liver in Response to Fasting

**DOI:** 10.3389/fphys.2020.00208

**Published:** 2020-03-18

**Authors:** Xiaofang Geng, Jianlin Guo, Lu Zhang, Jiyao Sun, Xiayan Zang, Zhigang Qiao, Cunshuan Xu

**Affiliations:** ^1^Henan Key Laboratory of Immunology and Targeted Therapy, School of Laboratory Medicine, Xinxiang Medical University, Xinxiang, China; ^2^State Key Laboratory Cultivation Base for Cell Differentiation Regulation, College of Life Sciences, Henan Normal University, Xinxiang, China; ^3^College of Fisheries, Henan Normal University, Xinxiang, China

**Keywords:** Chinese giant salamander, fasting, liver, metabolism, proteome

## Abstract

Chinese giant salamander *Andrias davidianus* has strong tolerance to starvation. Fasting triggers a complex array of adaptive metabolic responses, a process in which the liver plays a central role. Here, a high-throughput proteomic analysis was carried out on liver samples obtained from adult *A. davidianus* after 3, 7, and 11 months of fasting. As a result, the expression levels of 364 proteins were significantly changed in the fasted liver. Functional analysis demonstrated that the expression levels of key proteins involved in fatty acid oxidation, tricarboxylic acid cycle, gluconeogenesis, ketogenesis, amino acid oxidation, urea cycle, and antioxidant systems were increased in the fasted liver, especially at 7 and 11 months after fasting. In contrast, the expression levels of vital proteins involved in pentose phosphate pathway and protein synthesis were decreased after fasting. We also found that fasting not only activated fatty acid oxidation and ketogenesis-related transcription factors PPARA and PPARGC1A, but also activated gluconeogenesis-related transcription factors FOXO1, HNF4A, and KLF15. This study confirms the central role of lipid and acetyl-CoA metabolism in *A. davidianus* liver in response to fasting at the protein level and provides insights into the molecular mechanisms underlying the metabolic response of *A. davidianus* liver to fasting.

## Introduction

Groundwater biotopes are characterized by poor and discontinuous food supplies. The organisms faced with reduced food intake have evolved efficient physiological and metabolic adaptations to extend their survival (Issartel et al., 2010). Several hypogean species are able to survive for long periods of food deprivation—nearly 1 year in invertebrates, and up to several years in cave fishes and salamanders (Hervant et al., 1999; Hervant et al., 2001). A study report proposed that the hypogean species had a lower metabolic rate, higher amounts of energy reserves (glycogen, triglycerides, and proteins) and reduction in utilization rates than epigean ones, enabling them to fuel energy metabolism for much longer without food (Hervant and Renault, 2002; Issartel et al., 2010).

The liver is the central organ for metabolic activity in vertebrates and is also an important storage depot for energy reserve (glycogen and lipids). Urodele amphibians not only have large amounts of glycogen in hepatocytes, but also have larger amount of lipids than other vertebrates (Bizjak Mali et al., 2013). Hepatic fat serves as energy reserve in many vertebrates including fishes and amphibians during starvation (Groom et al., 2013). The utilization of the energy reserves varies in different species with successive periods of glycogen, lipid, and finally lipid–protein-dominant catabolism. For hypogean salamander *Proteus anguinus* that can tolerate fasting for between 18 and 96 months, its liver glycogen was decreased by 50%, lipids by 25%, and proteins by 35% after 18 months of starvation (Hervant et al., 2001; Bizjak Mali et al., 2013). Reduced hepatocyte size, scarce lipid droplets, and numerous mitochondria and peroxisomes were observed in the hepatocytes of *P. anguinus* after 18 months of starvation (Bizjak Mali et al., 2013). For the frog *Rana esculenta*, its liver glycogen concentration decreased by 95% after 18 months of starvation at 20°C (Grably and Piery, 1981). For the South African clawed toad *Xenopus laevis*, its liver glycogen levels were reduced by 80–90% after 12 months of starvation at 20°C (Merkle and Hanke, 1988). For other vertebrates, several studies showed that genes involved in ketogenesis, gluconeogenesis, and fatty acid beta-oxidation, were up-regulated in liver of chicken after 16 h of starvation (Desert et al., 2008), in liver of mice after 24 h of starvation (Kinouchi et al., 2018; Rennert et al., 2018), in liver of fishes after 21 days of starvation (Drew et al., 2008; Qian et al., 2016). Compared to other vertebrates, the hypogean salamanders exhibited remarkable resistance to long periods of food deprivation, this may be due to the remarkable accumulation of lipid and glycogen deposits in the liver, and glycogen level maintenance during food deprivation.

The giant salamanders are aquatic urodeles including Cryptobranchidae, Amphiumidae, Sirenidae, and Proteidae. The Cryptobranchidae include the hellbender, Japanese giant salamander, and Chinese giant salamander. They are largely water-breathers with reduced lung function. The lungs can function during hypoxia events, but routine gas exchange is aquatic, and almost entirely integumentary (Ultsch, 2012). Chinese giant salamander *Andrias davidianus* is the largest extant amphibian in the world and the only species of cryptobranchidae in China. It lives and breeds in large hill streams, normally in forested areas of central and southern China (Turvey et al., 2019). Wild species is threatened with extinction due to habitat loss, water pollution, and over-hunting, and thus is listed as a class II protected species of China and in the Appendix I of Convention on International Trade of Endangered Species (CITES, 2014). Over the past decade, the increased number of the artificially cultivated *A. davidianus* provide the potential resource for scientific research (He et al., 2018). However, owing to little genomic data was available previously for *A. davidianus*, it has hindered the understanding of molecular mechanisms underlying stress response and physiological adaptation of *A. davidianus*. Currently, complete transcriptome analysis reports of *A. davidianus* (Geng et al., 2017; Huang et al., 2017) pave the way for research on physiological adaptations to extreme environments. Giant salamanders can survive for long periods without food up to several years. However, it is unclear what molecular mechanisms underlie their survival. Therefore, to characterize the physiological and metabolic response of *A. davidianus* to prolonged fasting, we investigated protein expression changes in the liver from adult *A. davidianus* after 3, 7, and 11 months of fasting by iTRAQ-coupled RPLC-MS/MS. This study lays the foundation for understanding better how aquatic amphibians to survive in their food limited habitats.

## Materials and Methods

### Animals, Sample Preparation

Adult healthy female Chinese giant salamanders weighing about 2 kg, were obtained from an artificial breeding farm at Chongqing Kui Xu Biotechnology Incorporated Company in Chongqing, China. The giant salamanders were maintained in aerated freshwater tanks at 20°C under weak lighting conditions and fed daily with diced silver carp. The giant salamanders were acclimatized to laboratory conditions for 2 weeks prior to fasting. A total of 20 salamanders were randomly divided into 4 groups with 5 salamanders per group: three fasting groups and one normal control group. The salamanders in the fasting groups were subjected to fasting for 3, 7, and 11 months. The salamanders were heavily anesthetized by MS-222 and sacrificed by decapitation and liver tissues were collected from control group and fasting groups.

Then, protein extraction was performed according to a method described by Isaacson et al. (2006) with some modification. The liver tissues were ground into power with liquid nitrogen and precipitated by cooled acetone containing 10% TCA. It was then centrifuged by 1,5000 *g* for 15 min at 4°C and dried by vacuum freeze dryer. The deposit was suspended in cold phenol extraction buffer, and an equal volume of phenol saturated with Tris-HCl (pH 7.5) was added and centrifuged at 5000 *g* for 30 min at 4°C to collect the upper phenolic phase. Next, cold 0.1 M ammonium acetate in methanol was added for precipitation followed by centrifugation at 1,0000 *g* for 10 min at 4°C. Then, the deposit was washed by cold methanol and acetone and centrifuged at 1,0000 *g* for 10 min at 4°C. The dried deposit was dissolved in lysis solution at 30°C for 1 h, and centrifuged by 1,5000 *g* for 30 min. The supernatant was collected and protein concentration in the extracts were determined by the BCA method (Smith et al., 1985).

### Protein Digestion and iTRAQ Labeling

A total of 100 μg proteins from each sample were reduced and alkylated as described in the iTRAQ protocol (Applied Biosystems), followed by digestion with 50 ng/μL sequencing-grade trypsin solution at 37°C overnight. The digested peptides were dried by vacuum centrifugation. The peptides from control, 3, 7, and 11 months of fasting samples were labeled with 117, 118, 119, and 121 iTRAQ tags, respectively, according to the manufacturer’s protocol (Applied Biosystems, United States). The labeled samples were pooled and vacuum dried.

### RPLC-MS/MS Analysis

The dried sample was re-suspended in 100 μL buffer A (10 mM KH_2_PO_4_ pH 3.0, 25% acetonitrile), and separated on the Agilent 1200 HPLC System (Agilent). The parameters of RPLC were set as follows: analytical guard column (4.6 mm × 12.5 mm, 5 μm); narrow-bore (2.1 mm × 150 mm, 5 μm) with 215 and 280 nm UV detection; flow rate of 0.3 mL/min using a non-linear binary gradient starting with buffer A and transitioning to buffer B (10 mM KH_2_PO_4_ pH 3.0, 500 mM KCl, 25% acetonitrile). Each segment was collected at 4.5 min interval between 8 and 52 min. A total of 10 segments were collected and dried by a vacuum freeze dryer.

The online Nano-RPLC was employed on the Eksigent nanoLC-Ultra 2D System (AB SCIEX). The dried peptides from each segment were re-suspended in Nano-RPLC buffer A (0.1% formic acid, 2% acetonitrile) and loaded on C18 nanoLC trap column (100 μm × 3 cm, C18, 3 μm, 150 Å) and washed by Nano-RPLC buffer A at 2 μL/min for 10 min. An elution gradient of 5–35% acetonitrile (0.1% formic acid) run for 70 min was used on an analytical ChromXP C18 column (75 μm × 15 cm, C18, 3 μm 120 Å) with spray tip. Data acquisition was performed with a Triple TOF 5600 System (AB SCIEX, United States) fitted with a Nanospray III source (AB SCIEX, United States) and a pulled quartz tip as the emitter (New Objectives, United States). Data were acquired at an ion spray voltage of 2.5 kV, curtain gas of 30 psi, nebulizer gas of 5 psi, and an interface heater temperature of 150°C. For information dependent acquisition (IDA), survey scans were acquired at 250 ms, and as many as 35 product ion scans were collected if they exceeded a threshold of 150 counts/s with a 2+ to 5+ charge-state. The total cycle time was fixed to 2.5 s. A rolling collision energy setting was applied to all precursor ions for collision-induced dissociation (CID). Dynamic exclusion was set to 1/2 of peak width (18 s). The mass spectrometry proteomics data have been deposited to the ProteomeXchange Consortium via the PRIDE (Perez-Riverol et al., 2019) partner repository with the dataset identifier PXD014924.

### Data Analysis

Data was processed with Protein Pilot Software v. 5.0 (AB SCIEX, United States) against the protein database obtained from transcriptome data of *A. davidianus* using the Paragon algorithm (Shilov et al., 2007). The following search parameters were utilized to analyze MS/MS data: trypsin as the digestion enzyme with a maximum of two missed cleavage allowed, fixed modifications of Carbamidomethyl (C) and iTRAQplex modification (K and N-terminus), variable modifications of Oxidation (M), peptide mass tolerance of ±20 ppm, fragment mass tolerance of ±0.1 Da, and peptide false discovery rate (FDR) ≤0.01.

The quantification of iTRAQ labeled peptides was carried out based on reporter ion intensity using Proteome Discoverer 1.4 software. Only unused score >1.3 and unique peptide ≥1 were utilized to determine protein quantification. The frequency distribution histogram was developed to analyze iTRAQ quantitative data (Cox and Mann, 2008). Firstly, protein ratio was calculated as the relative expression level in fasting group to that in control group. Next, the *P*-value for log protein ratios was calculated using previously published method (Cox and Mann, 2008; Geng et al., 2016). Based on the above analysis, fold change >1.5 and *P*-value <0.05 were the selection criteria for the significantly changed proteins.

### Bioinformatics Analysis

To characterize the expression patterns of the proteins, Cluster 3.0/TreeView was utilized for hierarchical clustering of the differentially expressed proteins in the liver samples after fasting (Eisen et al., 1998). DAVID database was used to perform functional and pathway enrichment analyses of the differentially expressed proteins according to a modified Fisher’s exact test in combination with FDR method as described in detail previously (Huang da et al., 2009). In addition, the differentially expressed proteins were analyzed by Ingenuity Pathway Analysis (IPA) version 9.0 software for transcription regulators, canonical pathways and biological functions (Kramer et al., 2014).

### Western Blot Analysis

Antibodies raised against salamander proteins were not available, thus mammalian antibodies were used in this study. Briefly, 60 μg of proteins were separated by 12% SDS-PAGE and transferred to a nitrocellulose membrane. The membranes were incubated with rabbit anti-ADH1, anti-FBP1, anti-GOT2, and rabbit anti-YWHAE (Bioss, 1:1000) overnight at 4°C. The membrane was incubated with horseradish peroxidase (HRP)-conjugated secondary goat anti-rabbit IgG (Sigma, 1:2500). Finally, the protein band was visualized with Amersham enhanced chemiluminescence (ECL) substrates. Exposure imaging was performed on the ImageQuant LAS 4000 Chemiluminescence Imager (GE Healthcare), and the band density was measured using ImageQuant TL software (GE Healthcare). β-actin (Sigma, 1:1000) was used as an internal reference.

## Results

### Quantitative Proteomics Analysis Revealed Alterations in Liver Proteins in Fasted *Andrias davidianus*

**FIGURE 1:**
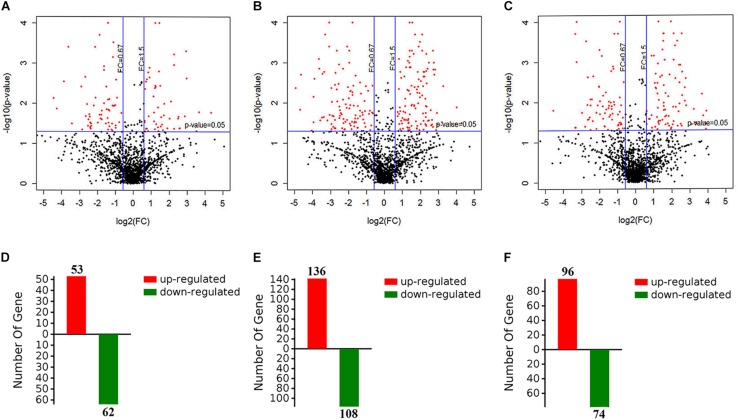
Screening of differentially expressed proteins in the fasted liver. **(A–C)** The volcano plot showing protein expression changes after 3, 7, and 11 months of fasting. **(D–F)** The number of differentially expressed proteins after 3, 7, and 11 months of fasting.

To evaluate the metabolic response of *A. davidianus* liver to fasting, iTRAQ coupled with LC-MS/MS technology was employed to assess proteome changes in the liver after 0 (control), 3, 7, and 11 months of fasting. A total of 1820 potential proteins were identified based on the screening criteria of unused score >1.3 and unique peptide ≥1. Based on the screening criteria of fold change >1.5 and *P*-value <0.05, 364 differentially expressed proteins were identified in the fasted liver (Supplementary Table S1), and the results are displayed by a volcano plot (Figure 1). Compared to control, more proteins had their levels changed after 7 months of fasting than 3 or 11 months of fasting.

### Western Blot Analysis

**FIGURE 2:**
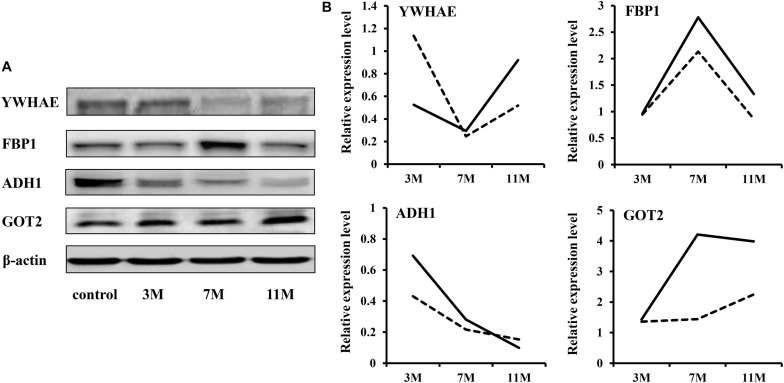
Western blot validation. **(A)** Protein expression levels were detected by western blot. β-Actin served as the internal reference. **(B)** Comparison of relative protein levels detected by iTRAQ and western blot. The solid line represents the iTRAQ data and the dotted line represents the western blot data.

To validate the iTRAQ results, four abundantly expressed proteins were randomly selected and subjected to western blot: 14-3-3 epsilon (YWHAE), fructose-1,6-bisphosphatase 1 (FBP1), alcohol dehydrogenase 1 (ADH1), and aspartate aminotransferase 2 (GOT2). The results showed that ADH1 was significantly down-regulated during fasting; YWHAE was significantly down-regulated after 7 months of fasting; FBP1 was significantly up-regulated after 7 months of fasting (Figure 2). Up-regulation expression of GOT2 was detected only after 11 months of fasting by western blot, but both after 7 and 11 months of fasting by iTRAQ, maybe due to that there is great randomness for acquiring fragmentation spectra of a precursor ion in the Triple TOF 5600. Overall, the protein expression trends between western blot and proteomic analysis were basically similar, suggesting that the results of iTRAQ were reliable.

### Clustering of Protein Expression Patterns in the Liver of the Fasted *Andrias davidianus*

**FIGURE 3:**
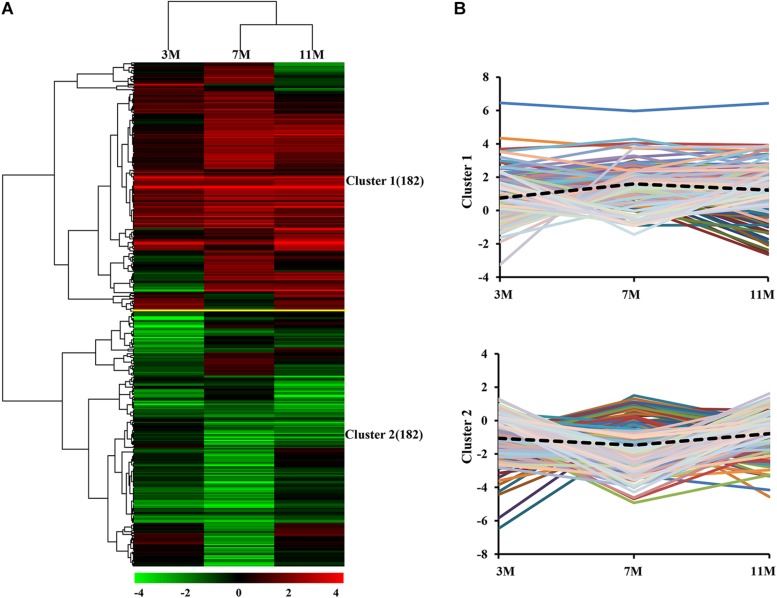
Global comparison of protein expression patterns in the fasted liver. **(A)** Hierarchical clustering of expression patterns of differentially expressed proteins. Red and green colors denote a higher and lower expression level than the control, respectively. **(B)** Average expression patterns in each cluster. Dotted line represents average fold changes of proteins in each cluster.

Dynamic expression patterns of differentially expressed proteins in the liver of *A. davidianus* after 3, 7, and 11 months of fasting were categorized by K-means clustering, and the results showed that 364 differentially expressed proteins were categorized into two clusters (Figure 3A). Cluster 1 contained proteins which showed an increasing trend, and the average expression level of 182 proteins in the liver after 7 and 11 months of fasting was higher than that at 3 months of fasting (Figure 3B). Cluster 2 contained proteins which showed a decreasing trend and the average expression level of 182 proteins in the liver after 7 months of fasting was lower than that at 3 and 11 months of fasting (Figure 3B).

### Functional and Pathway Enrichment Analysis of Differentially Expressed Proteins in the Liver of the Fasted *Andrias davidianus*

GO and KEGG pathway enrichment analyses of differentially expressed proteins were carried out by DAVID database. Up-regulated expression proteins in cluster 1 were significantly enriched in fatty acid beta-oxidation, tricarboxylic acid cycle, gluconeogenesis, urea cycle, glycogen catabolic process, and amino acid metabolism (Table 1). Among them, the expression of glycogen phosphorylase (PYGL), a key enzyme involved in glycogenolysis, was significantly increased after 3 and 7 months of fasting. Fructose 1,6-bisphosphatase (FBP1) and phosphoenolpyruvate carboxykinase (PCK1), key enzymes involved in gluconeogenesis, were significantly up-regulated after 7 and 11 months of fasting. Acyl CoA dehydrogenase (ACAD9, ACADL, and IVD) and 3-hydroxyacyl-CoA dehydrogenase (HADHA and HADHB) that participate in fatty acid beta-oxidation, were significantly up-regulated after 7 and 11 months of fasting. The expression of citrate synthase, a key enzyme involved in tricarboxylic acid cycle, was significantly enhanced after 7 and 11 months of fasting. The expression of hydroxymethylglutaryl coenzyme A synthetase (HMGCS1), a key enzyme involved in ketogenesis, was increased after 11 months of fasting. Aspartate aminotransferases (GOT1 and GOT2) involved in malate-aspartate shuttle, were significantly up-regulated after 7 and 11 months of fasting. The expression of urea cycle enzymes was increased after 7 and 11 months of fasting, such as argininosuccinate synthase (ASS1), ornithine carbamoyltransferase (OTC), and arginase (ARG1). The expression of cytochrome c oxidase (COX4I1), an electron transport chain terminal oxidase, was enhanced after 7 and 11 months of fasting. The expression levels of antioxidant enzymes were increased after 7 and 11 months of fasting, such as superoxide dismutase (SOD1 and SOD2), thioredoxin peroxidase (PRDX3) and glutathione peroxidase (GPX4).

**TABLE 1:** Function enrichment analysis of differentially expressed proteins.

**Enriched biological functions**	***P*-value**	**Genes**
**Cluster 1**		
Fatty acid beta-oxidation	5.88E-04	ACOX1, IVD, ACAD9, ACADL, ACAT1, HADHA, HADHB (7)
Tricarboxylic acid cycle	1.50E-03	ACO2, CS, MDH2, FH, MDH1 (4)
Carbohydrate metabolic process	1.60E-03	GLA, PYGL, NAGA, HEXB, UGDH, MDH2, MDH1 (7)
Lipid homeostasis	2.02E-03	ACOX1, IVD, ACAD9, ACADL (4)
Urea cycle	2.16E-03	ARG1, ASS1, OCT (3)
Gluconeogenesis	1.06E-02	GPI, TPI1, FBP1, PCK1 (4)
Cellular amino acid metabolic process	2.00E-02	GOT2, GOT1, OCT (3)
Glycogen catabolic process	2.43E-02	PYGL, AGL (2)
**Cluster 2**		
Protein folding	1.50E-06	HSP90AB1, P4HB, HSP90B1, CCT5, TXNDC5, CCT8, PDIA6, CCT2, CCT6A, CALR (10)
Translation	2.78E-04	RPS18, RPL6, RRBP1, RPL13A, SLC25A5, RPL3, SLC25A1, RPL24, RPS2, RPS3 (10)
Response to endoplasmic reticulum stress	4.14E-04	P4HB, EIF2S1, TXNDC5, PDIA6 (4)
Glycolytic process	1.22E-03	ALDOA, ALDOB, PKM, PGK1, ENO1 (5)
Pentose-phosphate shunt	3.28E-03	G6PD, TALDO1, PGD (3)

Down-regulated expression proteins in cluster 2 were significantly enriched in protein folding, translation, response to endoplasmic reticulum stress, glycolytic process, and pentose-phosphate shunt (Table 1). Among them, the expression of pyruvate kinase (PKM), a key enzyme involved in the glycolytic pathway, was significantly decreased after 3 and 11 months fasting. 6-Phosphogluconate dehydrogenase (PGD) and glucose-6-phosphate dehydrogenase (G6PD), the key enzymes of pentose phosphate pathway, were significantly down-regulated after 3 and 7 months of fasting. The decreased expression of translational elongation factor (EEF2), threonine tRNA ligase (TARS), and most ribosomal proteins was found in the fasted *A. davidianus* liver. The expression levels of chaperone proteins were reduced in the fasted *A. davidianus* liver, such as HSP90AB1 and HSP90B1.

**FIGURE 4:**
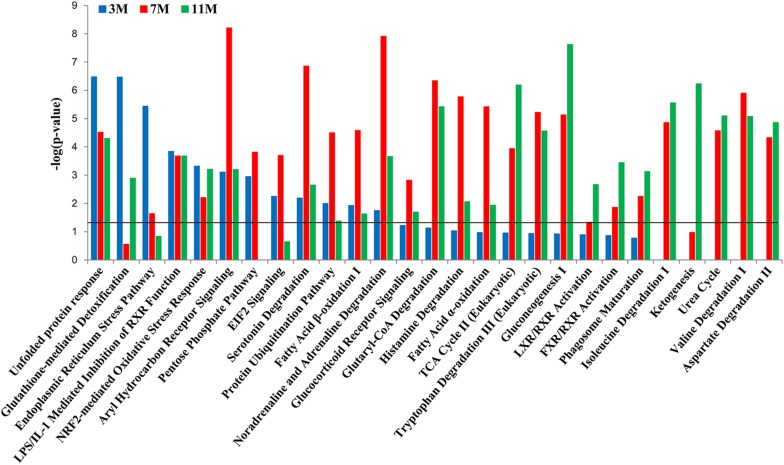
Pathway enrichment analysis of differentially expressed proteins in the fasted liver. Dark line represents the threshold of *P* < 0.05.

To further clarify which signaling pathways played important roles in the liver of *A. davidianus* after fasting, IPA analysis was carried out to connect differentially expressed proteins with canonical pathways. The results of pathway analysis showed that glutathione-mediated detoxification and endoplasmic reticulum stress pathway were significantly enriched after 3 months of fasting; protein ubiquitination pathway and fatty acid oxidation were significantly enriched after 7 months of fasting; while citric acid cycle, gluconeogenesis, and ketogenesis were significantly enriched after 11 months of fasting (Figure 4). Moreover, pentose phosphate pathway was only enriched after 3 and 7 months of fasting, whereas amino acid metabolism (isoleucine degradation I, valine degradation I, and aspartate degradation II) were enriched after 7 and 11 months of fasting (Figure 4).

### Activation Analysis of Upstream Regulators and Biofunctions in the Liver of the Fasted *Andrias davidianus*

**FIGURE 5:**
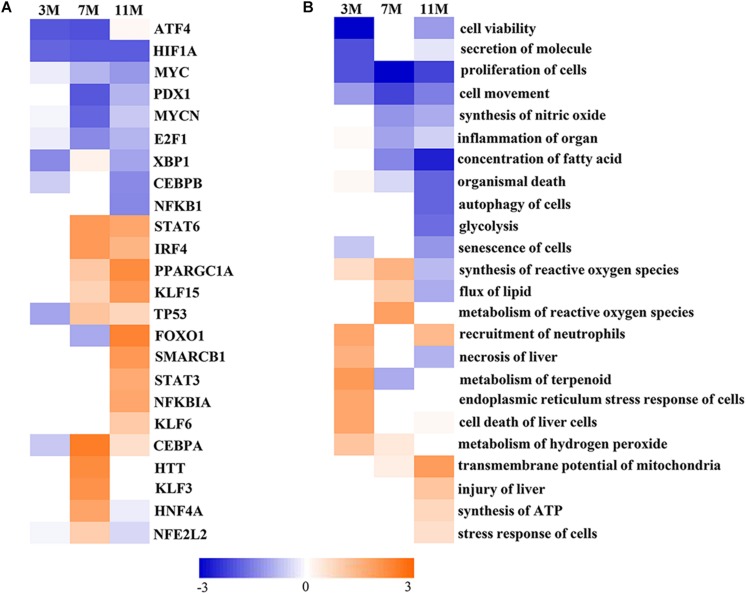
Activated or inhibited transcription factors and biological functions in the fasted liver. **(A)** Upstream transcription factors. **(B)** Biological functions. Jacinth and blue colors represent activation and inhibition of upstream transcription factor or biofunction, respectively.

Upstream Regulator Analysis in IPA is a novel function which can predict which transcriptional regulators are involved and whether they are likely activated or inhibited by analyzing linkage to differentially expressed proteins through coordinated expression. The results of Upstream Regulator Analysis demonstrated that transcription factors CCAAT-enhanced binding protein α (C/EBPα) and hepatocyte nuclear factor 4α (HNF4α) were likely activated after 7 months of fasting, and Krüppel-like factor 5 (KLF15), forkhead box protein FOXO1 and peroxisome proliferator-activated receptor γ coactivator 1α (PGC-1α) were likely activated after 11 months of fasting, whereas transcription factors MYCN and E2F1 that participate in protein synthesis were likely inhibited in the fasted liver (Figure 5A). Downstream Effects Analysis in IPA identifies downstream biological functions that are expected to be increased or decreased based on the gene expression changes. The results of Downstream Effects Analysis showed that necrosis and death of liver cells as well as endoplasmic reticulum stress response of cells were likely increased after 3 months of fasting; flux of lipid and synthesis of reactive oxygen species (ROS) were likely increased after 7 months of fasting; transmembrane potential of mitochondrial and synthesis of ATP were likely increased after 11 months of fasting (Figure 5B).

## Discussion

Hypogean salamanders are able to survive for several years without food, and they can adjust their metabolism to food deprivation by utilizing metabolites stored during times when food is abundant (Hervant et al., 2001). The metabolic reserves and their utilization vary with species. The metabolic response of animals to fasting is primarily coordinated by the liver. The molecular mechanisms that underlie their survival remain unclear. Although transcriptome analysis of mouse liver (Bauer et al., 2004; Li et al., 2006; Sokolovic et al., 2008) and chicken liver (Desert et al., 2008) in response to fasting has been reported, no investigation has explored the metabolic response of Urodelan amphibian liver to prolonged fasting. Therefore, this study examined the expression levels of proteins in the liver of *A. davidianus* after 3, 7, and 11 months of fasting. We found that the expression levels of proteins associated with fatty acid β-oxidation, amino acid metabolism, tricarboxylic acid cycle, gluconeogenesis, ketogenesis, and antioxidant system were up-regulated in the fasted *A. davidianus* liver.

### Up-Regulation of Liver Gluconeogenesis After Prolonged Fasting

At the early stages of fasting, hepatic glycogen degradation is enough to provide glucose to the extrahepatic tissue, but as the fasting continues, gluconeogenesis becomes more pronounced. The body mainly relies on gluconeogenesis to maintain a relatively constant blood glucose concentration. In this study, the expression levels of key proteins (FBP1 and PCK1) that participate in gluconeogenesis were found to be increased in the liver of *A. davidianus* after prolonged fasting. Transcription factors (FOXO1, HNF4α, KLF15, and C/EBPα) were likely activated in the liver after prolonged fasting by Upstream Regulator Analysis in IPA. FOXO1 is a key transcription factor that regulates hepatic gluconeogenesis. Under fasting conditions, FOXO1 expression is regulated by the coactivator p300 (Wondisford et al., 2014). FOXO1 synergizes with HNF4α to activate the expression of the gluconeogenesis rate-limiting enzyme G6Pase (Hirota et al., 2008; Dankel et al., 2010; Jitrapakdee, 2012). During fasting, KLF15 not only promotes gluconeogenesis by inducing expression of gluconeogenesis-related genes (Teshigawara et al., 2005; Takashima et al., 2010), but also induces alanine aminotransferase gene expression, thereby increasing amino acid-derived gluconeogenesis precursors supply, and hence gluconeogenesis (Gray et al., 2007). Under fasting conditions, C/EBPα induces the expression of the pyruvate carboxylase gene and promotes gluconeogenesis (Louet et al., 2010). These results indicate that liver gluconeogenesis was enhanced to maintain blood glucose levels of *A. davidianus* after prolonged fasting.

Studies have reported that the activity of key enzymes of glycolysis and lipogenesis was reduced in the *Sparus aurata* liver after 22 days of fasting (Meton et al., 1999). This study also found that the expression levels of key proteins (PKM, PGD, and G6PD) that modulate glycolysis and pentose phosphate pathway were decreased in the liver of *A. davidianus* after 3 and 7 months of fasting, revealing the decrease of glycolysis and pentose phosphate pathway in the fasted *A. davidianus*. The pentose phosphate pathway provides a variety of materials for biosynthetic metabolism, such as NADPH for fatty acid and cholesterol synthesis. These results indicate that biosynthesis was inhibited to save energy for the *A. davidianus* after prolonged fasting.

### Fatty Acid β-Oxidation and Ketogenesis Are Up-Regulated During Prolonged Fasting

Fasting inhibits lipogenesis and promotes fat mobilization, resulting in β-oxidation of fatty acids to provide energy for the body (Goldstein and Hager, 2015). This study found that the expression levels of key proteins that regulate fatty acid β-oxidation, tricarboxylic acid cycle, ketogenesis, and electron transport chain, were enhanced after 7 and 11 months of fasting. Downstream Effects Analysis by IPA predicted that the function of fatty acid oxidation and transmembrane potential of mitochondrial was likely enhanced after 7 and 11 months of fasting while fatty acid synthesis was likely inhibited after 11 months of fasting. These results confirm that the liver synthesizes large amount of ketone bodies from acetyl-CoA through β-oxidation of fatty acids to fuel extrahepatic tissues during prolonged fasting (Cahill, 2006; Newman and Verdin, 2014). In addition, transcription factor PPARα was likely activated in the liver of *A. davidianus* after prolonged fasting by Upstream Regulator Analysis in IPA. The activated PPARα by fasting promoted the expression of fatty acid oxidation and ketogenesis-related genes in synergy with other fasting-related transcription factors, such as CREB3L3 (Nakagawa et al., 2016), p300 (Zhang et al., 2016), and PGC-1α (Rodgers and Puigserver, 2007). These results indicate that the enhanced fatty acid oxidation is needed to provide energy for the *A. davidianus* after prolonged fasting and can also explain why *A. davidianus* can survive for long periods of food deprivation.

### Prolonged Fasting Is Accompanied by Increase of Proteolysis and Inhibition of Protein Synthesis

In the situation of limited energy supply, the organisms adapt by adjusting the rate of protein synthesis and degradation. A study showed that protein synthesis in the endoplasmic reticulum was significantly decreased and proteolysis was significantly increased in the liver of yellow croaker after 21 days of fasting (Qian et al., 2016). The increased expression of aspartate aminotransferase that participates in malate-aspartate shuttle in the fasted *A. davidianus* liver indicates that prolonged fasting induces tissue protein degradation, thus promoting gluconeogenesis of glucogenic amino acids. However, the reduced expression of ribosomal proteins, EEF2, MYCN, and E2F1 in the fasted *A. davidianu* liver may be involved in decreased protein synthesis function. Overall, prolonged fasting is accompanied by the increase of proteolysis and inhibition of protein synthesis in the *A. davidianu* liver.

Under stress conditions, molecular chaperones prevent protein aggregation and promote refolding of denatured proteins (Young et al., 2004). The expression levels of chaperone proteins were found to be reduced in the fasted *A. davidianus* liver, indicating that the decreased protein folding will help the organism to save energy under fasting conditions. Once protein folding is inhibited, the amount of misfolded proteins increases, resulting in ROS synthesis and unfolded protein response (UPR) (Goldberg, 2003). Collectively, it can be concluded that fasting inhibits protein folding in the *A. davidianus* liver, thereby inducing UPR.

During prolonged fasting, a large amount of NH4^+^ is produced by combined deamination in the liver, thereby generating urea through ornithine circulation. The up-regulation expression of vital proteins modulating urea cycle and arginine synthesis in the fasted *A. davidianus* liver indicates that amino acid oxidation and urea cycle were enhanced after prolonged fasting. Taken together, this study first reports that fasting induces amino acid oxidation and urea cycle in the amphibian liver.

### Prolonged Fasting Up-Regulates ROS Production and Antioxidant System

Oxidative stress can arise from overproduction of ROS by fatty acid β-oxidation and is defined as an imbalance between ROS production and antioxidant defense system. During fasting, many animals enhance their antioxidant defenses to cope with oxidative stress (Moreira et al., 2016). For example, lipid peroxidation and oxidative stress were strengthened in the liver of 72 h-fasted rats (Sorensen et al., 2006); the expression levels of antioxidant enzymes were up-regulated in the liver of 24 h-fasted mice (Sokolovic et al., 2008); antioxidant defense mechanism was activated in the liver of fish after 5 weeks of fasting (Morales et al., 2004). We also found that the expression levels of antioxidant enzymes were up-regulated in the liver of *A. davidianus* after prolonged fasting, due to that antioxidant defense system must minimize the levels of most harmful ROS. From these results, it can be concluded that the antioxidant defense system was enhanced to reduce oxidative damage caused by ROS overproduction, thus improving the tolerance of *A. davidianus* to prolonged fasting.

## Conclusion

**FIGURE 6:**
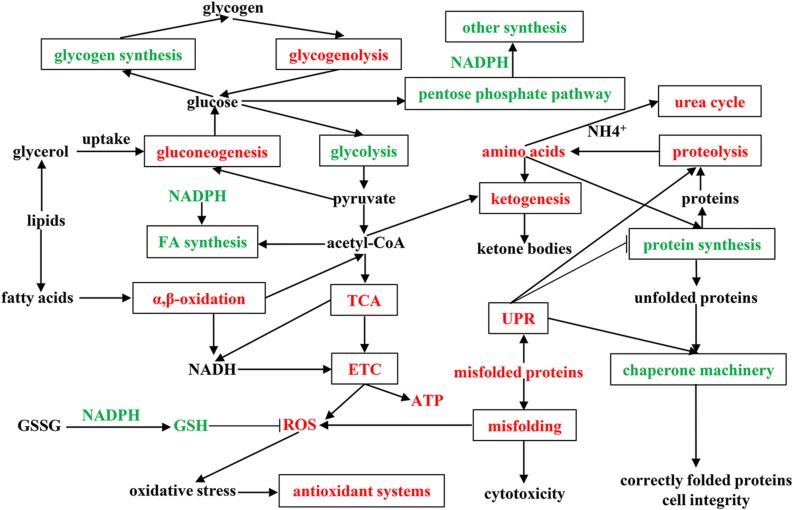
Molecular network for proteins in *A. davidianus* liver in response to long-term fasting. Red color represents up-regulation, green color represents down-regulation.

To fully understand the physiological and metabolic response of *A. davidianus* liver to prolonged fasting, a molecular network was established according to our results (Figure 6). The expression changes of proteins in the *A. davidianus* liver during long-term fasting was stage-dependent and closely related to fuel distribution. The expression levels of proteins involved in fatty acid β-oxidation, protein decomposition, tricarboxylic acid cycle, gluconeogenesis, ketogenesis, and urea cycle were increased after 7 and 11 months of fasting, while the expression levels of proteins involved in protein synthesis were inhibited. The enhanced fatty acid β-oxidation produced a large amount of ROS during fasting, and the organisms must up-regulate antioxidant defense system to reduce oxidative damage caused by ROS overproduction, thus improving the tolerance of *A. davidianus* to prolonged fasting. These results confirm the central role of lipid metabolism in *A. davidianus* liver in the response to fasting.

## Limitations of the Study

One potential limitation of our study is that it was carried out in the liver, and further studies should be done in the small intestine and stomach which also play key roles during fasting in the future. Another potential limitation is that the study is lack of “refeeding group” and is out of consideration of the influence of aging in the experimental design. Further studies are needed to verify these results in the future.

## Data Availability Statement

The proteomic data generated in this study can be found in the Proteomics Identifications Database (accession number: PXD014924).

## Ethics Statement

The animal handling and sampling procedures were performed in strict accordance with the guidelines of the Animal Care and Use Committee of Henan Normal University and were approved by the Animal Care and Ethics Committee of Henan Normal University.

## Author Contributions

CX conceived the project. XG carried out most of the experiments. JG and XZ conducted sample preparation. LZ and JS assisted in data analysis. ZQ assisted in animal feeding. XG and CX wrote the manuscript. All authors approved the final version of the manuscript.

## Conflict of Interest

The authors declare that the research was conducted in the absence of any commercial or financial relationships that could be construed as a potential conflict of interest.

## References

[B1] Bauer M. Hamm A. C. Bonaus M. Jacob A. Jaekel J. Schorle H. (2004). Starvation response in mouse liver shows strong correlation with life-span-prolonging processes. *Physiol. Genomics* 17 230–244. 10.1152/physiolgenomics.00203.2003 14762175

[B2] Bizjak Mali L. Sepcic K. Bulog B. (2013). Long-term starvation in cave salamander effects on liver ultrastructure and energy reserve mobilization. *J. Morphol.* 274 887–900. 10.1002/jmor.20145 23625365

[B3] Cahill G. F. Jr. (2006). Fuel metabolism in starvation. *Annu. Rev. Nutr.* 26 1–22. 10.1146/annurev.nutr.26.061505.111258 16848698

[B4] Cox J. Mann M. (2008). MaxQuant enables high peptide identification rates, individualized p.p.b.-range mass accuracies and proteome-wide protein quantification. *Nat. Biotechnol.* 26 1367–1372. 10.1038/nbt.1511 19029910

[B5] Dankel S. N. Hoang T. Flageng M. H. Sagen J. V. Mellgren G. (2010). cAMP-mediated regulation of HNF-4alpha depends on the level of coactivator PGC-1alpha. *Biochim. Biophys. Acta* 1803 1013–1019. 10.1016/j.bbamcr.2010.05.008 20670916

[B6] Desert C. Duclos M. J. Blavy P. Lecerf F. Moreews F. Klopp C. (2008). Transcriptome profiling of the feeding-to-fasting transition in chicken liver. *BMC Genomics* 9:611. 10.1186/1471-2164-9-611 19091074 PMC2628918

[B7] Drew R. E. Rodnick K. J. Settles M. Wacyk J. Churchill E. Powell M. S. (2008). Effect of starvation on transcriptomes of brain and liver in adult female zebrafish (Danio rerio). *Physiol. Genomics* 35 283–295. 10.1152/physiolgenomics.90213.2008 18728227 PMC2585019

[B8] Eisen M. B. Spellman P. T. Brown P. O. Botstein D. (1998). Cluster analysis and display of genome-wide expression patterns. *Proc. Natl. Acad. Sci. U.S.A.* 95 14863–14868. 10.1073/pnas.95.25.14863 9843981 PMC24541

[B9] Geng X. Chang C. Zang X. Sun J. Li P. Guo J. (2016). Integrative proteomic and microRNA analysis of the priming phase during rat liver regeneration. *Gene* 575(2 Pt 1), 224–232. 10.1016/j.gene.2015.08.066 26341052

[B10] Geng X. Li W. Shang H. Gou Q. Zhang F. Zang X. (2017). A reference gene set construction using RNA-seq of multiple tissues of Chinese giant salamander. *Andrias davidianus*. *Gigascience* 6 1–7. 10.1093/gigascience/gix006 28204480 PMC5467019

[B11] Goldberg A. L. (2003). Protein degradation and protection against misfolded or damaged proteins. *Nature* 426 895–899. 10.1038/nature02263 14685250

[B12] Goldstein I. Hager G. L. (2015). Transcriptional and chromatin regulation during fasting - the genomic era. *Trends Endocrinol. Metab.* 26 699–710. 10.1016/j.tem.2015.09.005 26520657 PMC4673016

[B13] Grably S. Piery Y. (1981). Weight and tissue changes in long term starved frogs *Rana esculenta*. *Comp. Biochem. Physiol.* 69 683–688. 10.1016/0300-9629(81)90156-0

[B14] Gray S. Wang B. Orihuela Y. Hong E. G. Fisch S. Haldar S. (2007). Regulation of gluconeogenesis by Kruppel-like factor 15. *Cell Metab.* 5 305–312. 10.1016/j.cmet.2007.03.002 17403374 PMC1892530

[B15] Groom D. J. Kuchel L. Richards J. G. (2013). Metabolic responses of the South American ornate horned frog (Ceratophrys ornata) to estivation. *Comp. Biochem. Physiol. B Biochem. Mol. Biol.* 164 2–9. 10.1016/j.cbpb.2012.08.001 22902863

[B16] He D. Zhu W. M. Zeng W. Lin J. Ji Y. Wang Y. (2018). Nutritional and medicinal characteristics of Chinese giant salamander (*Andrias davidianus*) for applications in healthcare industry by artificial cultivation: a review. *Food Sci.Hum. Wellness* 7 1–10. 10.1016/j.fshw.2018.03.001

[B17] Hervant F. Mathieu J. Barre H. (1999). Comparative study on the metabolic responses of subterranean and surface-dwelling amphipods to long-term starvation and subsequent refeeding. *J. Exp. Biol.* 202 (Pt 24), 3587–3595. 10574735 10.1242/jeb.202.24.3587

[B18] Hervant F. Mathieu J. Durand J. (2001). Behavioural, physiological and metabolic responses to long-term starvation and refeeding in a blind cave-dwelling (*Proteus anguinus*) and a surface-dwelling (Euproctus asper) salamander. *J. Exp. Biol.* 204 (Pt 2), 269–281. 11136613 10.1242/jeb.204.2.269

[B19] Hervant F. Renault D. (2002). Long-term fasting and realimentation in hypogean and epigean isopods: a proposed adaptive strategy for groundwater organisms. *J. Exp. Biol.* 205 (Pt 14), 2079–2087. 12089211 10.1242/jeb.205.14.2079

[B20] Hirota K. Sakamaki J. Ishida J. Shimamoto Y. Nishihara S. Kodama N. (2008). A combination of HNF-4 and Foxo1 is required for reciprocal transcriptional regulation of glucokinase and glucose-6-phosphatase genes in response to fasting and feeding. *J. Biol. Chem.* 283 32432–32441. 10.1074/jbc.M806179200 18805788

[B21] Huang Y. Xiong J. L. Gao X. C. Sun X. H. (2017). Transcriptome analysis of the Chinese giant salamander (*Andrias davidianus*) using RNA-sequencing. *Genom. Data* 14 126–131. 10.1016/j.gdata.2017.10.005 29159068 PMC5675895

[B22] Huang da W. Sherman B. T. Lempicki R. A. (2009). Systematic and integrative analysis of large gene lists using DAVID bioinformatics resources. *Nat. Protoc.* 4 44–57. 10.1038/nprot.2008.211 19131956

[B23] Isaacson T. Damasceno C. M. Saravanan R. S. He Y. Catala C. Saladie M. (2006). Sample extraction techniques for enhanced proteomic analysis of plant tissues. *Nat. Protoc.* 1 769–774. 10.1038/nprot.2006.102 17406306

[B24] Issartel J. Voituron Y. Guillaume O. Clobert J. Hervant F. (2010). Selection of physiological and metabolic adaptations to food deprivation in the Pyrenean newt Calotriton asper during cave colonisation. *Comp. Biochem. Physiol. A Mol. Integr. Physiol.* 155 77–83. 10.1016/j.cbpa.2009.10.002 19818868

[B25] Jitrapakdee S. (2012). Transcription factors and coactivators controlling nutrient and hormonal regulation of hepatic gluconeogenesis. *Int. J. Biochem. Cell Biol.* 44 33–45. 10.1016/j.biocel.2011.10.001 22004992

[B26] Kinouchi K. Magnan C. Ceglia N. Liu Y. Cervantes M. Pastore N. (2018). Fasting imparts a switch to alternative daily pathways in liver and muscle. *Cell Rep.* 25 3299.e6–3314.e6. 10.1016/j.celrep.2018.11.077 30566858 PMC6433478

[B27] Kramer A. Green J. Pollard J. Jr. Tugendreich S. (2014). Causal analysis approaches in ingenuity pathway analysis. *Bioinformatics* 30 523–530. 10.1093/bioinformatics/btt703 24336805 PMC3928520

[B28] Li R. Y. Zhang Q. H. Liu Z. Qiao J. Zhao S. X. Shao L. (2006). Effect of short-term and long-term fasting on transcriptional regulation of metabolic genes in rat tissues. *Biochem. Biophys. Res. Commun.* 344 562–570. 10.1016/j.bbrc.2006.03.155 16620784

[B29] Louet J. F. Chopra A. R. Sagen J. V. An J. York B. Tannour-Louet M. (2010). The coactivator SRC-1 is an essential coordinator of hepatic glucose production. *Cell Metab.* 12 606–618. 10.1016/j.cmet.2010.11.009 21109193 PMC3024581

[B30] Merkle S. Hanke W. (1988). Long-term starvation in *Xenopus laevis* Daudin. I. Effects on general metabolism. *Comp. Biochem. Physiol.* 89 719–730. 10.1016/0305-0491(88)90314-8 2901321

[B31] Meton I. Mediavilla D. Caseras A. Canto E. Fernandez F. Baanante I. V. (1999). Effect of diet composition and ration size on key enzyme activities of glycolysis-gluconeogenesis, the pentose phosphate pathway and amino acid metabolism in liver of gilthead sea bream (*Sparus aurata*). *Br. J. Nutr.* 82 223–232. 10.1017/s0007114599001403 10655969

[B32] Morales A. E. Perez-Jimenez A. Hidalgo M. C. Abellan E. Cardenete G. (2004). Oxidative stress and antioxidant defenses after prolonged starvation in *Dentex dentex* liver. *Comp. Biochem. Physiol. C Toxicol. Pharmacol.* 139 153–161. 10.1016/j.cca.2004.10.008 15556078

[B33] Moreira D. C. Venancio L. P. R. Sabino M. Hermes-Lima M. (2016). How widespread is preparation for oxidative stress in the animal kingdom? *Comp. Biochem. Physiol. A Mol. Integr. Physiol.* 200 64–78. 10.1016/j.cbpa.2016.01.023 26851497

[B34] Nakagawa Y. Satoh A. Tezuka H. Han S. I. Takei K. Iwasaki H. (2016). CREB3L3 controls fatty acid oxidation and ketogenesis in synergy with PPARalpha. *Sci. Rep.* 6 39182. 10.1038/srep39182 27982131 PMC5159891

[B35] Newman J. C. Verdin E. (2014). Ketone bodies as signaling metabolites. *Trends Endocrinol. Metab.* 25 42–52. 10.1016/j.tem.2013.09.002 24140022 PMC4176946

[B36] Perez-Riverol Y. Csordas A. Bai J. Bernal-Llinares M. Hewapathirana S. Kundu D. J. (2019). The PRIDE database and related tools and resources in 2019: improving support for quantification data. *Nucleic Acids Res.* 47 D442–D450. 10.1093/nar/gky1106 30395289 PMC6323896

[B37] Qian B. Xue L. Huang H. (2016). Liver Transcriptome Analysis of the Large Yellow Croaker (*Larimichthys crocea*) during Fasting by Using RNA-Seq. *PLoS One* 11:e0150240. 10.1371/journal.pone.0150240 26967898 PMC4788198

[B38] Rennert C. Vlaic S. Marbach-Breitruck E. Thiel C. Sales S. Shevchenko A. (2018). The diurnal timing of starvation differently impacts murine hepatic gene expression and lipid metabolism - a systems biology analysis using self-organizing maps. *Front. Physiol.* 9:1180. 10.3389/fphys.2018.01180 30271348 PMC6146234

[B39] Rodgers J. T. Puigserver P. (2007). Fasting-dependent glucose and lipid metabolic response through hepatic sirtuin 1. *Proc. Natl. Acad. Sci. U.S.A.* 104 12861–12866. 10.1073/pnas.0702509104 17646659 PMC1937557

[B40] Shilov I. V. Seymour S. L. Patel A. A. Loboda A. Tang W. H. Keating S. P. (2007). The Paragon Algorithm, a next generation search engine that uses sequence temperature values and feature probabilities to identify peptides from tandem mass spectra. *Mol. Cell Proteomics* 6 1638–1655. 10.1074/mcp.t600050-mcp200 17533153

[B41] Smith P. K. Krohn R. I. Hermanson G. T. Mallia A. K. Gartner F. H. Provenzano M. D. (1985). Measurement of protein using bicinchoninic acid. *Anal. Biochem.* 150 76–85. 10.1016/0003-2697(85)90442-7 3843705

[B42] Sokolovic M. Sokolovic A. Wehkamp D. Ver Loren Van Themaat E. De Waart D. R. Gilhuijs-Pederson L. A. (2008). The transcriptomic signature of fasting murine liver. *BMC Genomics* 9:528. 10.1186/1471-2164-9-528 18990241 PMC2588605

[B43] Sorensen M. Sanz A. Gomez J. Pamplona R. Portero-Otin M. Gredilla R. (2006). Effects of fasting on oxidative stress in rat liver mitochondria. *Free Radic. Res.* 40 339–347. 10.1080/10715760500250182 16517498

[B44] Takashima M. Ogawa W. Hayashi K. Inoue H. Kinoshita S. Okamoto Y. (2010). Role of KLF15 in regulation of hepatic gluconeogenesis and metformin action. *Diabetes* 59 1608–1615. 10.2337/db09-1679 20393151 PMC2889759

[B45] Teshigawara K. Ogawa W. Mori T. Matsuki Y. Watanabe E. Hiramatsu R. (2005). Role of Kruppel-like factor 15 in PEPCK gene expression in the liver. *Biochem. Biophys. Res. Commun.* 327 920–926. 10.1016/j.bbrc.2004.12.096 15649433

[B46] Turvey S. T. Marr M. M. Barnes I. Brace S. Tapley B. Murphy R. W. (2019). Historical museum collections clarify the evolutionary history of cryptic species radiation in the world’s largest amphibians. *Ecol. Evol.* 9 10070–10084. 10.1002/ece3.5257 31624538 PMC6787787

[B47] Ultsch G. R. (2012). Metabolism, gas exchange, and acid-base balance of giant salamanders. *Biol. Rev. Camb. Philos. Soc.* 87 583–601. 10.1111/j.1469-185X.2011.00211.x 22151821

[B48] Wondisford A. R. Xiong L. Chang E. Meng S. Meyers D. J. Li M. (2014). Control of Foxo1 gene expression by co-activator P300. *J. Biol. Chem.* 289 4326–4333. 10.1074/jbc.M113.540500 24379407 PMC3924295

[B49] Young J. C. Agashe V. R. Siegers K. Hartl F. U. (2004). Pathways of chaperone-mediated protein folding in the cytosol. *Nat. Rev. Mol. Cell Biol.* 5 781–791. 10.1038/nrm1492 15459659

[B50] Zhang Z. N. Gong L. Lv S. Li J. Tai X. Cao W. (2016). SIK2 regulates fasting-induced PPARalpha activity and ketogenesis through p300. *Sci. Rep.* 6 23317. 10.1038/srep23317 26983400 PMC4794759

