# Supplementary material for: Differential Proteomic Analysis of Chinese Giant Salamander Liver in Response to Fasting
Source: Front Physiol. 2020 Mar 18;11:208. doi: 10.3389/fphys.2020.00208 (PMC7093600; doi:10.3389/fphys.2020.00208)
Supplement: Supplementary file 1 [file Table_1.DOC]

**Table S1 Differentially expressed proteins in the liver of the fasted *Andrias davidianus***

| **ID** | **Unused** | **Total** | **% Cov** | **Peptides**  **(95%)** | **Genes** | **3M** | **P value** | **7M** | **P value** | **11M** | **P value** |
| --- | --- | --- | --- | --- | --- | --- | --- | --- | --- | --- | --- |
| CL5936.Contig2_All | 18.53 | 25.18 | 54.7 | 24 | A2M | 0.545 | 0.708 | 2.377 | 0.086 | 2.965 | 0.016 |
| Unigene9522_All | 56.64 | 58.31 | 33 | 39 | A2ML1 | 0.297 | 0.026 | 0.855 | 0.846 | 1.077 | 0.701 |
| Unigene6214_All | 19.28 | 19.52 | 32 | 12 | AADAC | 1.486 | 0.485 | 5.546 | 0.001 | 2.559 | 0.029 |
| Unigene740_All | 3.43 | 3.6 | 11.9 | 2 | ABCE1 | 2.831 | 0.004 | 2.831 | 0.004 | 4.613 | 0.003 |
| Unigene5374_All | 42.14 | 42.2 | 55.8 | 28 | ACAD9 | 0.904 | 0.849 | 2.109 | 0.115 | 2.270 | 0.024 |
| Unigene8389_All | 20.11 | 20.21 | 41 | 14 | ACADL | 1.445 | 0.173 | 3.373 | 0.000 | 2.109 | 0.003 |
| Unigene34927_All | 18.48 | 18.64 | 33.3 | 12 | ACADSB | 0.157 | 0.087 | 2.831 | 0.012 | 0.912 | 0.743 |
| Unigene1718_All | 59.11 | 59.21 | 78.9 | 88 | ACAT1 | 1.191 | 0.033 | 4.446 | 0.000 | 5.105 | 0.000 |
| Unigene3638_All | 2.32 | 2.38 | 57.7 | 1 | ACBD7 | 7.379 | 0.021 | 3.733 | 0.348 | 2.704 | 0.465 |
| CL7941.Contig1_All | 44.77 | 44.87 | 41.1 | 27 | ACO1 | 2.443 | 0.033 | 0.982 | 0.815 | 2.377 | 0.090 |
| Unigene1756_All | 51.6 | 51.64 | 51.5 | 45 | ACO2 | 1.213 | 0.657 | 2.334 | 0.001 | 4.246 | 0.000 |
| Unigene31554_All | 35.95 | 36 | 48.4 | 29 | ACOX2 | 4.055 | 0.592 | 3.467 | 0.695 | 5.970 | 0.007 |
| Unigene3948_All | 5.6 | 7.08 | 14.3 | 4 | ACSBG2 | 0.011 | 0.045 | 0.441 | 0.287 | 0.592 | 0.414 |
| CL1309.Contig6_All | 16.62 | 16.66 | 50.7 | 14 | ACTN1 | 1.871 | 0.131 | 2.443 | 0.041 | 0.895 | 0.930 |
| CL1309.Contig7_All | 103.86 | 103.85 | 68.2 | 88 | ACTN4 | 0.511 | 0.041 | 1.077 | 0.904 | 0.421 | 0.001 |
| Unigene732_All | 40.02 | 40.25 | 60.3 | 26 | ACTR3 | 0.398 | 0.008 | 0.991 | 0.558 | 1.077 | 0.360 |
| Unigene2152_All | 24.14 | 24.23 | 23.3 | 14 | ADAR | 0.184 | 0.019 | 0.483 | 0.696 | 0.457 | 0.085 |
| Unigene758_All | 43.41 | 45.13 | 67 | 35 | ADH1 | 0.692 | 0.296 | 0.281 | 0.000 | 0.099 | 0.000 |
| CL8210.Contig2_All | 86.34 | 86.34 | 88.7 | 138 | ADH4 | 0.912 | 0.695 | 0.168 | 0.000 | 0.302 | 0.000 |
| Unigene1083_All | 17.41 | 17.57 | 39.5 | 13 | ADH5 | 5.649 | 0.024 | 9.728 | 0.004 | 3.162 | 0.308 |
| CL1419.Contig1_All | 31.92 | 31.95 | 58.3 | 27 | ADK | 1.459 | 0.372 | 2.249 | 0.017 | 1.057 | 0.879 |
| Unigene11455_All | 153.21 | 153.75 | 66.5 | 135 | AGL | 1.294 | 0.483 | 3.105 | 0.000 | 0.794 | 0.826 |
| Unigene68894_All | 32.69 | 32.73 | 56 | 55 | AGXT | 1.127 | 0.815 | 3.532 | 0.244 | 4.325 | 0.001 |
| Unigene28523_All | 55.05 | 55.46 | 70 | 54 | AGXT2 | 1.306 | 0.753 | 0.194 | 0.000 | 1.787 | 0.207 |
| Unigene637_All | 57.57 | 57.67 | 63.9 | 60 | AHCY | 1.000 | 0.444 | 4.286 | 0.000 | 0.955 | 0.681 |
| Unigene2583_All | 5.6 | 5.73 | 21.2 | 3 | AK3 | 0.698 | 0.428 | 0.146 | 0.043 | 0.291 | 0.097 |
| Unigene842_All | 12.98 | 14.8 | 42.4 | 8 | AKR1B1 | 0.191 | 0.031 | 0.698 | 0.201 | 0.099 | 0.043 |
| CL823.Contig2_All | 39.57 | 39.59 | 84.9 | 49 | ALAD | 1.306 | 0.067 | 4.018 | 0.000 | 0.643 | 0.695 |
| Unigene39497_All | 133.71 | 133.7 | 90.3 | 268 | ALB | 1.077 | 0.129 | 4.571 | 0.000 | 4.529 | 0.000 |
| Unigene10267_All | 68.84 | 68.85 | 78.5 | 86 | ALDH1B1 | 1.787 | 0.002 | 0.102 | 0.000 | 1.406 | 0.378 |
| Unigene12277_All | 66.93 | 67.01 | 53.8 | 37 | ALDH1L1 | 0.724 | 0.078 | 0.360 | 0.012 | 0.667 | 0.045 |
| Unigene4689_All | 26.01 | 35.82 | 38.3 | 22 | ALDH1L2 | 3.598 | 0.117 | 4.966 | 0.018 | 3.311 | 0.091 |
| CL8711.Contig1_All | 38.94 | 39.15 | 63.6 | 29 | ALDH6A1 | 1.959 | 0.303 | 0.175 | 0.001 | 1.500 | 0.605 |
| Unigene3625_All | 42.08 | 42.11 | 56.1 | 46 | ALDH7A1 | 1.406 | 0.146 | 7.047 | 0.000 | 2.938 | 0.004 |
| Unigene74668_All | 39.27 | 39.36 | 53.1 | 37 | ALDH8A1 | 1.959 | 0.726 | 3.162 | 0.012 | 0.802 | 0.040 |
| Unigene1734_All | 66.23 | 68.58 | 61.4 | 89 | ALDH9A1 | 1.038 | 0.798 | 3.733 | 0.000 | 0.560 | 0.037 |
| Unigene652_All | 39.45 | 39.59 | 72.8 | 54 | ALDOA | 1.097 | 0.726 | 0.692 | 0.988 | 0.586 | 0.036 |
| Unigene646_All | 33.24 | 37.64 | 84.8 | 157 | ALDOB | 0.619 | 0.698 | 2.014 | 0.220 | 0.398 | 0.013 |
| CL4040.Contig2_All | 51.49 | 51.49 | 72.7 | 37 | ALLC | 0.912 | 0.145 | 2.679 | 0.001 | 2.032 | 0.141 |
| Unigene56851_All | 29.71 | 29.8 | 50.4 | 27 | ALN | 0.855 | 0.191 | 3.945 | 0.000 | 0.236 | 0.012 |
| CL3971.Contig2_All | 4.89 | 5.01 | 13.8 | 3 | ALOX5 | 1.570 | 0.028 | 1.318 | 0.067 | 1.191 | 0.082 |
| Unigene4018_All | 8.32 | 8.67 | 22.5 | 6 | AMACR | 3.404 | 0.052 | 2.938 | 0.127 | 3.598 | 0.023 |
| Unigene7715_All | 24.02 | 24.02 | 41.1 | 20 | AMT | 1.706 | 0.392 | 5.012 | 0.001 | 4.571 | 0.056 |
| Unigene753_All | 4.48 | 6.67 | 28.4 | 6 | ANP32B | 0.479 | 0.881 | 2.858 | 0.038 | 2.291 | 0.278 |
| CL4521.Contig4_All | 20.88 | 20.93 | 22.8 | 15 | ANPEP | 0.196 | 0.032 | 1.202 | 0.889 | 1.472 | 0.870 |
| Unigene518_All | 34.15 | 36.23 | 62.7 | 31 | ANXA1 | 0.597 | 0.333 | 0.270 | 0.005 | 0.575 | 0.058 |
| Unigene39038_All | 26.57 | 28.91 | 56.3 | 22 | ANXA13 | 0.938 | 0.201 | 0.331 | 0.002 | 0.223 | 0.018 |
| Unigene1328_All | 80.35 | 80.28 | 80.1 | 146 | ANXA4 | 1.019 | 0.292 | 1.923 | 0.051 | 0.172 | 0.000 |
| Unigene1040_All | 41.16 | 41.32 | 66.5 | 32 | ANXA5 | 0.752 | 0.454 | 0.227 | 0.011 | 0.802 | 0.290 |
| Unigene10790_All | 30.55 | 30.83 | 37.3 | 28 | AOC3 | 2.291 | 0.267 | 4.446 | 0.019 | 1.959 | 0.997 |
| Unigene8057_All | 39.55 | 39.63 | 38.4 | 30 | AOX1 | 1.355 | 0.746 | 2.606 | 0.042 | 0.384 | 0.010 |
| Unigene6980_All | 10.01 | 10.01 | 22.9 | 5 | APOA1BP | 1.600 | 0.945 | 5.546 | 0.028 | 2.512 | 0.260 |
| Unigene40959_All | 278.56 | 278.56 | 44 | 167 | APOB | 1.406 | 0.177 | 3.076 | 0.000 | 2.208 | 0.005 |
| Unigene6733_All | 81.28 | 81.35 | 92.9 | 200 | ARG1 | 0.832 | 0.331 | 4.018 | 0.000 | 1.738 | 0.026 |
| Unigene1003_All | 25.81 | 25.96 | 57.7 | 17 | ARPC2 | 0.445 | 0.187 | 0.402 | 0.129 | 0.254 | 0.010 |
| Unigene1291_All | 9.76 | 9.93 | 67.6 | 8 | ARPC5 | 0.711 | 0.968 | 0.334 | 0.031 | 1.028 | 0.783 |
| Unigene1354_All | 53.24 | 53.38 | 58.8 | 57 | ASL | 0.711 | 0.831 | 1.906 | 0.159 | 0.251 | 0.001 |
| Unigene664_All | 99.42 | 99.42 | 86.8 | 259 | ASS1 | 1.306 | 0.786 | 2.679 | 0.001 | 0.268 | 0.000 |
| CL509.Contig3_All | 27.81 | 29.91 | 53.7 | 61 | ASTL | 0.929 | 0.489 | 3.404 | 0.029 | 3.373 | 0.059 |
| CL388.Contig1_All | 29.08 | 29.13 | 27.4 | 18 | ATP1A1 | 0.263 | 0.350 | 0.506 | 0.053 | 0.177 | 0.012 |
| CL40.Contig2_All | 4.19 | 4.23 | 18.1 | 2 | ATP1B3 | 1.355 | 0.292 | 0.904 | 0.843 | 0.166 | 0.026 |
| CL4486.Contig4_All | 23.83 | 23.96 | 21.4 | 15 | ATP2A2 | 0.643 | 0.390 | 0.319 | 0.030 | 0.380 | 0.225 |
| Unigene680_All | 58.05 | 58.21 | 63.5 | 43 | ATP5A1 | 0.817 | 0.624 | 0.160 | 0.001 | 2.148 | 0.004 |
| Unigene556_All | 41.34 | 41.38 | 60.2 | 51 | ATP5B | 1.000 | 0.701 | 0.160 | 0.005 | 1.854 | 0.001 |
| CL4282.Contig2_All | 14.48 | 14.65 | 43.4 | 13 | ATP5C1 | 0.802 | 0.696 | 0.141 | 0.040 | 1.127 | 0.558 |
| Unigene790_All | 8.11 | 8.3 | 46.3 | 5 | ATP5H | 1.690 | 0.003 | 1.159 | 0.005 | 1.306 | 0.003 |
| Unigene769_All | 6.21 | 6.31 | 40.2 | 3 | ATP5L | 2.128 | 0.528 | 7.047 | 0.070 | 10.471 | 0.036 |
| CL8312.Contig2_All | 16.9 | 16.96 | 37 | 9 | ATP5O | 1.556 | 0.239 | 0.530 | 0.645 | 3.162 | 0.037 |
| Unigene774_All | 12.02 | 12.03 | 66.1 | 8 | B2M | 0.146 | 0.170 | 0.421 | 0.340 | 0.177 | 0.049 |
| Unigene1789_All | 8.29 | 8.42 | 28.5 | 5 | BCAP31 | 1.406 | 0.453 | 0.061 | 0.049 | 1.294 | 0.748 |
| CL5590.Contig1_All | 13.7 | 13.8 | 27.4 | 11 | BCHE | 1.959 | 0.680 | 4.875 | 0.034 | 4.169 | 0.036 |
| Unigene6429_All | 24.55 | 24.59 | 54.4 | 15 | BDH1 | 1.225 | 0.385 | 0.773 | 0.860 | 2.938 | 0.011 |
| Unigene2364_All | 39.88 | 39.98 | 70.5 | 42 | BHMT | 0.511 | 0.107 | 1.770 | 0.019 | 0.391 | 0.002 |
| Unigene6808_All | 5.39 | 5.81 | 27 | 4 | BLES03 | 2.679 | 0.049 | 2.559 | 0.082 | 1.542 | 0.491 |
| Unigene36008_All | 156.07 | 156.17 | 61.3 | 139 | C3 | 0.470 | 0.000 | 1.787 | 0.066 | 1.170 | 0.412 |
| Unigene44754_All | 34.16 | 34.63 | 18.4 | 20 | C4 | 1.318 | 0.828 | 2.051 | 0.044 | 1.159 | 0.681 |
| Unigene38214_All | 37.93 | 38.03 | 25.3 | 18 | C5 | 4.131 | 0.024 | 6.855 | 0.002 | 6.982 | 0.000 |
| Unigene807_All | 21.88 | 22.16 | 68.5 | 23 | CALR | 0.219 | 0.011 | 1.028 | 0.620 | 0.445 | 0.000 |
| Unigene580_All | 41.62 | 41.63 | 83.1 | 46 | CAPG | 0.759 | 0.384 | 0.263 | 0.000 | 0.773 | 0.402 |
| Unigene756_All | 12.69 | 12.9 | 41.3 | 8 | CAPNS1 | 0.340 | 0.493 | 0.322 | 0.025 | 0.603 | 0.628 |
| Unigene5738_All | 13.32 | 13.36 | 60.6 | 9 | CAT | 0.337 | 0.272 | 0.384 | 0.258 | 2.128 | 0.042 |
| Unigene2880_All | 23.4 | 23.43 | 68.1 | 13 | CBR1 | 0.189 | 0.039 | 0.129 | 0.034 | 0.597 | 0.278 |
| CL5994.Contig1_All | 32.51 | 32.52 | 60 | 20 | CCBL1 | 0.824 | 0.731 | 3.664 | 0.002 | 0.929 | 0.880 |
| Unigene831_All | 38 | 38.08 | 63.6 | 22 | CCT2 | 0.398 | 0.076 | 0.163 | 0.009 | 0.614 | 0.151 |
| Unigene659_All | 30.73 | 30.84 | 49.2 | 20 | CCT5 | 0.752 | 0.785 | 0.097 | 0.023 | 0.938 | 0.864 |
| Unigene686_All | 22.84 | 22.95 | 40.9 | 14 | CCT6A | 0.731 | 0.733 | 0.215 | 0.046 | 0.964 | 0.637 |
| CL113.Contig2_All | 40.04 | 40.08 | 50.4 | 23 | CCT8 | 1.107 | 0.464 | 0.244 | 0.004 | 1.486 | 0.486 |
| CL832.Contig2_All | 10.07 | 10.16 | 47.9 | 6 | CD8A | 0.069 | 0.003 | 1.000 | 0.579 | 0.421 | 0.079 |
| Unigene41513_All | 5.1 | 6.78 | 16.4 | 4 | CES2 | 0.581 | 0.030 | 1.331 | 0.495 | 0.946 | 0.899 |
| Unigene43321_All | 16.82 | 16.99 | 28.3 | 11 | CES3 | 0.200 | 0.109 | 1.000 | 0.711 | 0.042 | 0.016 |
| Unigene567_All | 27.36 | 27.4 | 80 | 47 | CFL1 | 0.982 | 0.972 | 0.134 | 0.000 | 0.847 | 0.315 |
| CL1675.Contig4_All | 8.09 | 8.24 | 33.5 | 8 | CIRBP | 0.603 | 0.625 | 0.091 | 0.023 | 0.128 | 0.018 |
| Unigene3809_All | 8 | 8 | 50 | 5 | CISD1 | 1.148 | 0.705 | 3.500 | 0.045 | 4.131 | 0.056 |
| Unigene1353_All | 28.41 | 28.42 | 78.8 | 25 | CLIC1 | 0.855 | 0.664 | 0.180 | 0.015 | 0.182 | 0.080 |
| Unigene18713_All | 29.93 | 30.02 | 46.6 | 20 | CMAS | 2.208 | 0.067 | 4.831 | 0.008 | 1.213 | 0.879 |
| Unigene1912_All | 10.07 | 10.08 | 41.8 | 5 | CMPK1 | 0.608 | 0.246 | 0.039 | 0.015 | 0.283 | 0.063 |
| Unigene6136_All | 4.74 | 4.81 | 8.1 | 3 | COL4A2 | 1.888 | 0.002 | 2.148 | 0.002 | 1.259 | 0.004 |
| Unigene1859_All | 45.71 | 46.86 | 55.5 | 62 | COL6A1 | 0.879 | 0.817 | 1.923 | 0.028 | 1.191 | 0.872 |
| CL514.Contig1_All | 242.14 | 242.06 | 54.6 | 225 | COL6A3 | 0.920 | 0.261 | 3.162 | 0.000 | 2.421 | 0.000 |
| Unigene25157_All | 93.42 | 93.46 | 32.5 | 60 | COL6A6 | 0.887 | 0.562 | 2.443 | 0.005 | 1.380 | 0.349 |
| Unigene1177_All | 16.46 | 16.56 | 12.1 | 10 | COPB1 | 1.837 | 0.021 | 1.660 | 0.017 | 1.472 | 0.021 |
| Unigene1478_All | 16.4 | 16.68 | 24 | 10 | COPB2 | 0.187 | 0.022 | 1.127 | 0.477 | 0.177 | 0.009 |
| Unigene715_All | 4.02 | 4.02 | 24.9 | 2 | COX4I1 | 0.511 | 0.036 | 5.152 | 0.003 | 5.445 | 0.003 |
| Unigene11514_All | 16.88 | 17 | 25.1 | 13 | CP | 0.384 | 0.030 | 1.019 | 0.926 | 0.904 | 0.688 |
| Unigene11782_All | 26.62 | 26.65 | 49.5 | 15 | CPOX | 0.409 | 0.327 | 3.908 | 0.006 | 5.297 | 0.001 |
| Unigene6910_All | 27.96 | 27.96 | 47.4 | 17 | CPQ | 1.202 | 0.767 | 5.012 | 0.002 | 1.977 | 0.183 |
| Unigene4662_All | 8.6 | 8.71 | 19.6 | 5 | CR1 | 0.603 | 0.103 | 0.673 | 0.092 | 0.581 | 0.036 |
| CL4820.Contig1_All | 12.07 | 12.08 | 38 | 7 | CTBS | 0.421 | 0.409 | 2.466 | 0.042 | 0.824 | 0.581 |
| Unigene10968_All | 39.36 | 39.89 | 57.5 | 32 | CTH | 0.445 | 0.032 | 0.120 | 0.000 | 0.366 | 0.023 |
| CL1330.Contig1_All | 9.3 | 9.45 | 30 | 11 | CTSD | 2.512 | 0.303 | 3.565 | 0.037 | 4.699 | 0.013 |
| Unigene2103_All | 15.84 | 15.95 | 44.6 | 15 | CTSK | 0.718 | 0.484 | 2.249 | 0.012 | 1.343 | 0.476 |
| CL7099.Contig3_All | 7.97 | 8.23 | 27.8 | 5 | CTSL1 | 1.117 | 0.056 | 2.911 | 0.011 | 1.854 | 0.022 |
| Unigene445_All | 18.14 | 18.19 | 64.6 | 39 | CYP1 | 1.138 | 0.657 | 0.395 | 0.016 | 0.319 | 0.006 |
| CL5009.Contig3_All | 24.72 | 25.62 | 35.6 | 15 | CYP2A13 | 0.179 | 0.026 | 0.090 | 0.000 | 0.402 | 0.015 |
| Unigene8173_All | 20.05 | 20.11 | 33.6 | 16 | CYP2D15 | 0.402 | 0.015 | 0.619 | 0.474 | 0.421 | 0.684 |
| Unigene9363_All | 15.49 | 17.09 | 36.2 | 10 | CYP2F3 | 0.201 | 0.194 | 0.955 | 0.632 | 1.528 | 0.017 |
| CL5009.Contig1_All | 16.24 | 21.64 | 26.5 | 11 | CYP2G1 | 0.879 | 0.366 | 0.871 | 0.371 | 0.506 | 0.039 |
| Unigene1200_All | 8.11 | 8.67 | 49.6 | 9 | DBI | 7.798 | 0.001 | 0.540 | 0.376 | 0.545 | 0.886 |
| CL4501.Contig2_All | 10.44 | 10.7 | 31.3 | 9 | DCN | 2.704 | 0.541 | 6.310 | 0.027 | 4.613 | 0.149 |
| CL791.Contig3_All | 15.53 | 15.64 | 28.8 | 15 | DDX5 | 0.242 | 0.119 | 0.219 | 0.045 | 0.745 | 0.587 |
| Unigene5726_All | 28.51 | 28.55 | 55.9 | 18 | DECR1 | 2.884 | 0.444 | 4.920 | 0.023 | 4.786 | 0.189 |
| Unigene3363_All | 26.23 | 26.24 | 41.2 | 18 | DLD | 3.162 | 0.248 | 6.730 | 0.001 | 5.649 | 0.036 |
| Unigene3537_All | 20.44 | 20.45 | 39.9 | 12 | DNASE2 | 0.817 | 0.770 | 6.730 | 0.438 | 5.395 | 0.011 |
| Unigene1889_All | 20.09 | 20.56 | 36.3 | 16 | DNPEP | 3.598 | 0.162 | 6.546 | 0.001 | 2.805 | 0.175 |
| CL7820.Contig2_All | 4.76 | 5.03 | 27.9 | 5 | DOM3Z | 0.347 | 0.032 | 0.449 | 0.302 | 0.185 | 0.009 |
| Unigene33537_All | 65.34 | 66.48 | 70.1 | 113 | DPYS | 1.019 | 0.506 | 2.559 | 0.005 | 0.302 | 0.040 |
| Unigene3291_All | 46.65 | 47.6 | 12.9 | 25 | DYNC1H1 | 0.437 | 0.440 | 0.413 | 0.074 | 0.322 | 0.015 |
| Unigene1947_All | 36.02 | 36.06 | 73.9 | 29 | ECH1 | 1.432 | 0.230 | 3.733 | 0.003 | 2.780 | 0.002 |
| Unigene3891_All | 19.02 | 19.64 | 48.4 | 21 | ECHDC2 | 0.483 | 0.498 | 1.803 | 0.034 | 0.545 | 0.777 |
| Unigene562_All | 14.03 | 14.11 | 45.4 | 10 | EEF1B2 | 1.629 | 0.813 | 0.198 | 0.043 | 1.019 | 0.361 |
| CL6954.Contig1_All | 64.43 | 64.8 | 55.9 | 47 | EEF2 | 0.231 | 0.000 | 0.102 | 0.000 | 0.511 | 0.000 |
| CL5508.Contig1_All | 46.8 | 47.62 | 40.5 | 27 | EHHADH | 0.625 | 0.467 | 0.429 | 0.018 | 1.294 | 0.949 |
| Unigene917_All | 24.14 | 24.23 | 54.9 | 14 | EIF2S1 | 0.136 | 0.004 | 0.334 | 0.061 | 0.492 | 0.659 |
| CL2340.Contig1_All | 81.19 | 81.3 | 85.5 | 99 | ENO1 | 1.306 | 0.014 | 0.441 | 0.026 | 0.686 | 0.021 |
| Unigene7315_All | 20.12 | 23.95 | 36 | 14 | EPHX1 | 0.283 | 0.088 | 1.820 | 0.013 | 0.497 | 0.161 |
| CL4219.Contig4_All | 49.34 | 49.4 | 48.3 | 34 | EPPK1 | 0.449 | 0.050 | 0.103 | 0.000 | 0.056 | 0.000 |
| Unigene1022_All | 6.19 | 6.3 | 43.3 | 5 | ERH | 0.299 | 0.025 | 0.136 | 0.104 | 1.803 | 0.325 |
| CL6803.Contig1_All | 31.21 | 31.34 | 55.2 | 20 | ERLIN2 | 1.117 | 0.834 | 1.644 | 0.434 | 3.467 | 0.035 |
| Unigene1565_All | 12.34 | 12.5 | 23.8 | 8 | ERP44 | 0.832 | 0.403 | 0.802 | 0.317 | 0.363 | 0.010 |
| Unigene2868_All | 14 | 14 | 29.6 | 8 | ESD | 0.895 | 0.518 | 4.131 | 0.003 | 1.170 | 0.566 |
| Unigene2101_All | 24.73 | 24.74 | 69.8 | 20 | ETFA | 0.738 | 0.695 | 0.291 | 0.222 | 2.559 | 0.002 |
| Unigene49636_All | 8.06 | 8.07 | 15 | 4 | F10 | 1.380 | 0.614 | 4.529 | 0.047 | 3.873 | 0.076 |
| Unigene636_All | 47.6 | 47.61 | 76.5 | 41 | F11 | 3.020 | 0.112 | 1.225 | 0.419 | 7.047 | 0.012 |
| Unigene57687_All | 17.67 | 17.74 | 92.1 | 110 | FABP1 | 0.724 | 0.463 | 0.673 | 0.109 | 0.196 | 0.002 |
| Unigene5790_All | 33.44 | 33.46 | 41.8 | 26 | FAH | 2.377 | 0.472 | 2.535 | 0.032 | 1.306 | 0.508 |
| Unigene3400_All | 22.29 | 22.38 | 55.9 | 16 | FAHD2 | 3.698 | 0.212 | 4.920 | 0.097 | 3.532 | 0.007 |
| CL6651.Contig2_All | 9.51 | 25.67 | 47.9 | 19 | FBP1 | 64.269 | 0.019 | 87.902 | 0.017 | 87.096 | 0.018 |
| CL1783.Contig1_All | 53.38 | 53.52 | 49.6 | 50 | FCGBP | 0.113 | 0.000 | 0.240 | 0.000 | 0.597 | 0.131 |
| CL5133.Contig1_All | 45.37 | 45.39 | 62.5 | 38 | FH | 1.318 | 0.770 | 4.325 | 0.001 | 2.938 | 0.013 |
| Unigene15989_All | 35.97 | 37.08 | 39.6 | 25 | FMO2 | 0.391 | 0.063 | 0.258 | 0.014 | 1.486 | 0.563 |
| CL1488.Contig2_All | 66.15 | 68.24 | 28.8 | 43 | FN1 | 0.377 | 0.046 | 1.028 | 0.590 | 0.745 | 0.257 |
| Unigene12108_All | 39.47 | 39.5 | 44.4 | 21 | FSCN1 | 0.402 | 0.122 | 0.281 | 0.045 | 0.160 | 0.002 |
| Unigene11551_All | 62.69 | 62.71 | 72 | 89 | FTCD | 4.325 | 0.009 | 7.112 | 0.002 | 6.026 | 0.002 |
| Unigene444_All | 18.78 | 18.93 | 52.8 | 17 | FTH1 | 0.530 | 0.276 | 1.629 | 0.111 | 13.428 | 0.022 |
| CL1757.Contig2_All | 42.56 | 42.59 | 56.2 | 29 | G6PD | 1.432 | 0.053 | 0.288 | 0.029 | 1.000 | 0.703 |
| Unigene14384_All | 35.81 | 35.88 | 38.2 | 21 | GBE1 | 1.225 | 0.868 | 0.182 | 0.021 | 0.619 | 0.633 |
| CL2448.Contig1_All | 18.05 | 21.61 | 34.2 | 14 | GBP1 | 0.169 | 0.011 | 0.406 | 0.098 | 0.350 | 0.049 |
| Unigene1162_All | 21.48 | 21.55 | 28.2 | 13 | GCAT | 2.831 | 0.235 | 2.679 | 0.409 | 6.668 | 0.004 |
| Unigene3945_All | 19.38 | 19.54 | 37.8 | 10 | GCDH | 0.575 | 0.329 | 0.177 | 0.012 | 0.311 | 0.019 |
| Unigene58099_All | 29.87 | 29.91 | 39 | 17 | GCKR | 2.489 | 0.187 | 2.704 | 0.006 | 2.355 | 0.328 |
| Unigene34447_All | 22.36 | 22.45 | 70.4 | 16 | GGT1 | 20.324 | 0.017 | 13.305 | 0.129 | 12.023 | 0.102 |
| Unigene9851_All | 8.08 | 10.14 | 30 | 9 | GLA | 0.592 | 0.354 | 6.310 | 0.035 | 3.532 | 0.640 |
| CL1457.Contig4_All | 10.86 | 11.63 | 39.8 | 12 | GNAI3 | 0.920 | 0.695 | 0.603 | 0.037 | 0.545 | 0.017 |
| Unigene1962_All | 22 | 22.87 | 48.8 | 13 | GNB2 | 0.855 | 0.520 | 0.649 | 0.134 | 0.357 | 0.014 |
| CL5063.Contig1_All | 46.87 | 46.89 | 45.3 | 39 | GNE | 0.847 | 0.672 | 0.391 | 0.021 | 0.363 | 0.013 |
| Unigene4936_All | 21.28 | 22.31 | 71 | 61 | GNMT | 1.514 | 0.157 | 6.668 | 0.002 | 2.535 | 0.064 |
| Unigene2685_All | 41.19 | 41.22 | 62.5 | 35 | GOT1 | 1.803 | 0.310 | 5.546 | 0.000 | 1.959 | 0.382 |
| Unigene995_All | 61.57 | 61.59 | 85.1 | 70 | GOT2 | 1.432 | 0.189 | 4.207 | 0.000 | 3.981 | 0.000 |
| CL5135.Contig3_All | 2.12 | 2.15 | 11.6 | 1 | GPHN | 1.600 | 0.004 | 1.754 | 0.005 | 1.445 | 0.004 |
| Unigene1035_All | 58.02 | 58.42 | 64.7 | 80 | GPI | 0.766 | 0.433 | 2.679 | 0.001 | 0.417 | 0.019 |
| Unigene44188_All | 35.51 | 35.62 | 64.9 | 76 | GRHPR | 1.486 | 0.727 | 5.105 | 0.012 | 3.565 | 0.134 |
| Unigene12471_All | 58.63 | 58.68 | 75.3 | 64 | GSS | 2.355 | 0.000 | 4.446 | 0.000 | 0.938 | 0.401 |
| Unigene668_All | 44.07 | 44.12 | 82.7 | 54 | GSTM5 | 2.780 | 0.000 | 1.097 | 0.336 | 0.745 | 0.971 |
| CL3930.Contig2_All | 22.13 | 22.3 | 52.3 | 11 | GSTO1 | 0.449 | 0.425 | 0.575 | 0.212 | 0.159 | 0.012 |
| Unigene597_All | 30.73 | 30.75 | 68.1 | 52 | GSTP1 | 2.377 | 0.004 | 1.393 | 0.013 | 1.500 | 0.019 |
| CL9031.Contig1_All | 39.06 | 41.05 | 66.3 | 70 | GSTT1 | 1.117 | 0.882 | 0.169 | 0.000 | 1.097 | 0.958 |
| Unigene2330_All | 29.16 | 29.25 | 68.7 | 29 | GSTZ1 | 4.831 | 0.001 | 0.661 | 0.340 | 3.105 | 0.016 |
| CL4381.Contig2_All | 13.42 | 14.16 | 26.3 | 9 | H1F0 | 0.497 | 0.011 | 0.033 | 0.004 | 0.105 | 0.000 |
| Unigene786_All | 43.58 | 43.61 | 61.2 | 40 | H2AFY2 | 0.363 | 0.014 | 0.151 | 0.000 | 0.724 | 0.275 |
| Unigene2876_All | 17.9 | 17.98 | 58.1 | 15 | HADH | 1.180 | 0.897 | 0.111 | 0.027 | 1.380 | 0.633 |
| CL2349.Contig2_All | 80.24 | 80.38 | 60.4 | 69 | HADHA | 1.191 | 0.173 | 1.282 | 0.790 | 3.467 | 0.000 |
| Unigene2188_All | 41.91 | 42 | 61.2 | 31 | HADHB | 1.355 | 0.459 | 1.271 | 0.998 | 6.252 | 0.000 |
| Unigene515_All | 28.22 | 28.28 | 78.9 | 95 | HBA | 0.492 | 0.285 | 5.297 | 0.001 | 6.730 | 0.000 |
| Unigene483_All | 19.87 | 22.08 | 68.3 | 146 | HBA2 | 0.413 | 0.784 | 3.221 | 0.004 | 2.884 | 0.000 |
| CL7002.Contig2_All | 28.35 | 28.39 | 63.7 | 63 | HBB1 | 0.268 | 0.166 | 3.532 | 0.000 | 3.873 | 0.000 |
| Unigene11938_All | 6.87 | 6.98 | 34.4 | 4 | HDHD2 | 1.871 | 0.001 | 2.466 | 0.001 | 2.355 | 0.001 |
| CL125.Contig3_All | 33.35 | 33.36 | 60.6 | 25 | HEXB | 0.380 | 0.405 | 3.733 | 0.000 | 3.192 | 0.006 |
| Unigene2594_All | 34.83 | 34.91 | 69.1 | 51 | HIBADH | 1.343 | 0.734 | 5.861 | 0.000 | 6.668 | 0.000 |
| CL1968.Contig2_All | 10.16 | 12.87 | 68.4 | 28 | HIST1H3B | 0.402 | 0.038 | 1.675 | 0.958 | 1.542 | 0.811 |
| Unigene506_All | 11.82 | 15.93 | 24.5 | 11 | HNRNPA3 | 0.474 | 0.061 | 0.308 | 0.012 | 0.619 | 0.024 |
| CL2296.Contig2_All | 24.12 | 24.29 | 50.5 | 18 | HNRNPAB | 0.353 | 0.201 | 0.082 | 0.001 | 0.938 | 0.228 |
| Unigene717_All | 16.68 | 20.72 | 35.4 | 20 | HNRNPH1 | 0.766 | 0.540 | 0.360 | 0.042 | 0.759 | 0.630 |
| CL4403.Contig1_All | 23.02 | 23.09 | 35.6 | 19 | HNRNPH3 | 0.291 | 0.052 | 0.129 | 0.046 | 0.711 | 0.417 |
| Unigene14018_All | 26.31 | 26.56 | 56.2 | 15 | HOGA1 | 0.139 | 0.046 | 0.809 | 0.421 | 0.608 | 0.463 |
| Unigene68959_All | 12.58 | 12.62 | 35.9 | 9 | HP | 0.094 | 0.032 | 1.542 | 0.553 | 1.159 | 0.966 |
| Unigene40438_All | 53.78 | 53.81 | 69.6 | 54 | HPD | 0.479 | 0.097 | 1.906 | 0.002 | 0.429 | 0.002 |
| CL7037.Contig2_All | 20.43 | 20.44 | 95 | 60 | HRSP12 | 1.213 | 0.304 | 5.970 | 0.015 | 2.535 | 0.277 |
| CL2156.Contig2_All | 26.75 | 26.77 | 47.3 | 36 | HSD11B1 | 0.545 | 0.282 | 1.854 | 0.002 | 0.887 | 0.803 |
| Unigene62460_All | 31.7 | 31.71 | 59.3 | 25 | HSD17B13 | 0.322 | 0.001 | 0.920 | 0.986 | 0.555 | 0.142 |
| CL4339.Contig1_All | 2.09 | 2.1 | 29.5 | 2 | HSD17B14 | 4.831 | 0.044 | 7.311 | 0.048 | 10.093 | 0.033 |
| CL5632.Contig3_All | 18.6 | 18.64 | 52 | 12 | HSD17B2 | 7.943 | 0.002 | 4.131 | 0.149 | 5.546 | 0.001 |
| CL3411.Contig1_All | 31.65 | 31.67 | 32.6 | 23 | HSD17B4 | 1.803 | 0.049 | 2.334 | 0.027 | 1.770 | 0.159 |
| CL1457.Contig3_All | 64.16 | 64.94 | 54.6 | 53 | HSP90AB1 | 0.773 | 0.039 | 0.466 | 0.009 | 0.920 | 0.162 |
| Unigene906_All | 54.87 | 60.07 | 48.6 | 47 | HSP90B1 | 0.353 | 0.004 | 0.161 | 0.000 | 0.863 | 0.403 |
| Unigene665_All | 71.91 | 80.47 | 71.8 | 77 | HSPA1 | 2.421 | 0.004 | 5.649 | 0.000 | 2.443 | 0.036 |
| CL3073.Contig4_All | 26.07 | 53.42 | 56.8 | 48 | HSPA2 | 1.906 | 0.215 | 2.148 | 0.026 | 1.138 | 0.301 |
| Unigene2136_All | 92.41 | 92.42 | 75.7 | 88 | HSPA5 | 0.461 | 0.001 | 0.242 | 0.000 | 0.887 | 0.189 |
| CL3073.Contig2_All | 35.61 | 70.95 | 63.7 | 73 | HSPA8 | 0.608 | 0.069 | 0.184 | 0.000 | 0.545 | 0.008 |
| Unigene1193_All | 58.53 | 58.55 | 73.6 | 58 | HSPD1 | 1.138 | 0.386 | 5.105 | 0.000 | 6.730 | 0.000 |
| Unigene2348_All | 16.12 | 16.23 | 78.4 | 18 | HSPE1 | 0.802 | 0.582 | 3.500 | 0.005 | 2.938 | 0.000 |
| CL5902.Contig1_All | 34.7 | 34.75 | 13.2 | 19 | HSPG2 | 3.342 | 0.090 | 6.138 | 0.037 | 1.803 | 0.953 |
| Unigene954_All | 48.61 | 49.05 | 50.3 | 37 | IDH2 | 0.879 | 0.508 | 0.051 | 0.000 | 0.766 | 0.185 |
| CL6210.Contig2_All | 40.41 | 40.77 | 61.8 | 56 | IGHE | 4.875 | 0.001 | 6.368 | 0.000 | 6.918 | 0.003 |
| Unigene6317_All | 24.53 | 24.57 | 41.7 | 18 | IVD | 0.466 | 0.191 | 4.571 | 0.002 | 1.871 | 0.432 |
| Unigene67099_All | 10.75 | 10.97 | 34 | 9 | IYD | 2.938 | 0.022 | 0.540 | 0.099 | 2.805 | 0.028 |
| CL8863.Contig1_All | 17.16 | 17.4 | 31.9 | 10 | KHDRBS1 | 0.196 | 0.037 | 0.483 | 0.203 | 1.432 | 0.092 |
| Unigene1122_All | 33.33 | 33.37 | 29.3 | 22 | KPNB1 | 0.938 | 0.953 | 0.353 | 0.042 | 0.614 | 0.482 |
| Unigene654_All | 47.7 | 49.73 | 65.9 | 46 | KRT19 | 1.629 | 0.663 | 1.941 | 0.288 | 4.571 | 0.000 |
| Unigene873_All | 7.28 | 7.39 | 48 | 7 | KRT7 | 0.142 | 0.096 | 0.066 | 0.007 | 0.413 | 0.180 |
| Unigene1206_All | 3.18 | 3.3 | 19.4 | 4 | LAMP3 | 0.052 | 0.014 | 1.585 | 0.538 | 1.038 | 0.989 |
| Unigene57098_All | 6.56 | 6.61 | 9.3 | 4 | LCAT | 1.038 | 0.164 | 1.259 | 0.084 | 1.528 | 0.036 |
| Unigene856_All | 37.58 | 37.71 | 64.6 | 33 | LDHA | 0.592 | 0.318 | 1.191 | 0.092 | 0.247 | 0.004 |
| Unigene28726_All | 8.16 | 8.18 | 49.6 | 7 | LGALS2 | 0.384 | 0.015 | 0.067 | 0.003 | 0.281 | 0.013 |
| Unigene7097_All | 7.95 | 8.1 | 23.6 | 6 | LHPP | 1.556 | 0.029 | 1.977 | 0.033 | 1.419 | 0.057 |
| Unigene1542_All | 16 | 16.81 | 34.7 | 11 | MAPK3 | 1.380 | 0.949 | 0.316 | 0.031 | 0.840 | 0.298 |
| Unigene778_All | 40.46 | 40.62 | 68.3 | 44 | MDH1 | 0.780 | 0.713 | 1.459 | 0.074 | 0.194 | 0.030 |
| Unigene642_All | 48.83 | 48.86 | 66.8 | 75 | MDH2 | 0.802 | 0.423 | 3.221 | 0.000 | 2.148 | 0.002 |
| Unigene60044_All | 14.15 | 14.27 | 47.2 | 11 | METTL7A | 6.081 | 0.042 | 3.532 | 0.043 | 14.588 | 0.036 |
| Unigene579_All | 8 | 8 | 35.4 | 5 | MGST3 | 11.482 | 0.049 | 5.105 | 0.152 | 14.997 | 0.048 |
| CL5501.Contig2_All | 9.93 | 10.01 | 18.7 | 5 | MMP18 | 1.028 | 0.938 | 0.268 | 0.011 | 1.086 | 0.864 |
| Unigene5402_All | 37.23 | 37.25 | 43.7 | 29 | MMP9 | 2.729 | 0.002 | 0.809 | 0.918 | 3.698 | 0.002 |
| CL8245.Contig1_All | 36.1 | 36.16 | 26.5 | 19 | MOV10 | 0.832 | 0.778 | 0.413 | 0.077 | 0.311 | 0.041 |
| Unigene58940_All | 80.09 | 80.27 | 60.4 | 146 | MPO | 2.249 | 0.029 | 7.516 | 0.000 | 2.805 | 0.001 |
| CL1597.Contig2_All | 12.75 | 12.79 | 41.1 | 11 | MPST | 5.152 | 0.273 | 0.366 | 0.321 | 3.981 | 0.044 |
| Unigene39019_All | 22.22 | 22.31 | 55.3 | 59 | MRC1 | 0.322 | 0.002 | 0.955 | 0.999 | 0.150 | 0.000 |
| Unigene625_All | 38 | 38.05 | 62.2 | 32 | MVP | 1.148 | 0.946 | 0.492 | 0.004 | 0.863 | 0.807 |
| Unigene1632_All | 11.29 | 11.42 | 30.1 | 9 | MYG1 | 0.631 | 0.936 | 2.992 | 0.077 | 3.105 | 0.023 |
| CL5726.Contig1_All | 16.37 | 16.56 | 38.1 | 16 | NAGA | 1.107 | 0.847 | 4.742 | 0.002 | 3.631 | 0.040 |
| Unigene605_All | 10.64 | 10.68 | 22.1 | 9 | NAP1L1 | 0.766 | 0.160 | 0.625 | 0.017 | 0.780 | 0.502 |
| Unigene1695_All | 24.92 | 25.86 | 39.9 | 17 | NARS | 1.706 | 0.383 | 0.221 | 0.029 | 0.938 | 0.301 |
| Unigene11392_All | 19.07 | 19.1 | 42.1 | 11 | NAT1 | 0.745 | 0.179 | 0.643 | 0.019 | 0.879 | 0.834 |
| CL4332.Contig2_All | 5.47 | 5.58 | 14.6 | 3 | NCF1 | 2.312 | 0.004 | 0.223 | 0.063 | 2.334 | 0.003 |
| CL3890.Contig6_All | 36.2 | 37.23 | 37 | 35 | NCL | 0.637 | 0.017 | 0.395 | 0.003 | 1.191 | 0.390 |
| Unigene2584_All | 7.43 | 7.52 | 51.6 | 5 | NHP2L1 | 4.699 | 0.079 | 1.393 | 0.645 | 6.668 | 0.010 |
| CL83.Contig1_All | 39.19 | 39.28 | 24.1 | 23 | NID1 | 3.281 | 0.020 | 3.192 | 0.012 | 4.325 | 0.000 |
| Unigene31387_All | 16.02 | 16.93 | 50.7 | 19 | NIPSNAP1 | 1.754 | 0.674 | 5.058 | 0.038 | 4.246 | 0.013 |
| Unigene3373_All | 30.49 | 30.53 | 69.2 | 26 | NIT2 | 0.780 | 0.943 | 2.655 | 0.020 | 0.938 | 0.834 |
| Unigene2734_All | 22.12 | 22.23 | 14.5 | 15 | NNT | 2.606 | 0.013 | 0.686 | 0.979 | 1.180 | 0.873 |
| CL7556.Contig2_All | 8.18 | 8.21 | 16.5 | 5 | NONO | 0.492 | 0.053 | 0.133 | 0.010 | 0.603 | 0.198 |
| Unigene1273_All | 12 | 12 | 22.2 | 6 | NT5C1A | 0.281 | 0.227 | 0.066 | 0.031 | 0.586 | 0.229 |
| CL5151.Contig2_All | 13.88 | 14.07 | 25.1 | 9 | OLFM4 | 0.146 | 0.140 | 0.679 | 0.514 | 0.286 | 0.007 |
| CL7344.Contig1_All | 44.9 | 44.95 | 68 | 69 | OTC | 1.837 | 0.374 | 3.281 | 0.000 | 1.225 | 0.844 |
| CL1564.Contig1_All | 8.02 | 9.81 | 6.4 | 6 | OVOS | 12.823 | 0.017 | 16.293 | 0.013 | 15.136 | 0.017 |
| Unigene557_All | 80.76 | 80.77 | 85.9 | 131 | P4HB | 0.227 | 0.000 | 1.393 | 0.996 | 0.550 | 0.000 |
| Unigene1189_All | 60.62 | 60.65 | 72.7 | 48 | PAICS | 1.995 | 0.133 | 3.133 | 0.001 | 0.501 | 0.078 |
| Unigene41916_All | 20.76 | 21.07 | 43.1 | 14 | PBLD | 2.249 | 0.017 | 4.699 | 0.002 | 1.148 | 0.758 |
| Unigene3339_All | 63.29 | 63.37 | 66.3 | 68 | PCK2 | 1.837 | 0.270 | 0.273 | 0.005 | 3.076 | 0.001 |
| CL4233.Contig1_All | 17.94 | 17.98 | 70.5 | 14 | PCMT1 | 1.294 | 0.362 | 4.488 | 0.004 | 3.281 | 0.022 |
| CL7942.Contig2_All | 2.65 | 2.75 | 4.2 | 3 | PCNT | 0.104 | 0.092 | 13.677 | 0.034 | 14.322 | 0.033 |
| CL336.Contig4_All | 31.37 | 31.47 | 63 | 48 | PDIA3 | 0.625 | 0.699 | 2.704 | 0.084 | 2.421 | 0.032 |
| Unigene3650_All | 66.7 | 66.82 | 54.7 | 52 | PDIA4 | 0.904 | 0.324 | 2.911 | 0.001 | 1.191 | 0.506 |
| Unigene1977_All | 36.39 | 38.3 | 49.7 | 38 | PDIA6 | 0.603 | 0.705 | 2.466 | 0.027 | 0.863 | 0.830 |
| Unigene1004_All | 27.85 | 27.96 | 40.9 | 22 | PGD | 0.380 | 0.000 | 1.722 | 0.050 | 0.492 | 0.051 |
| Unigene1251_All | 49.25 | 50.73 | 72.2 | 37 | PGK1 | 1.077 | 0.674 | 0.535 | 0.005 | 1.077 | 0.800 |
| CL8748.Contig2_All | 26.32 | 26.47 | 60.7 | 18 | PHB | 2.109 | 0.091 | 0.586 | 0.083 | 4.966 | 0.000 |
| Unigene1057_All | 52.32 | 52.43 | 58.3 | 42 | PHGDH | 1.138 | 0.053 | 0.698 | 0.249 | 0.196 | 0.000 |
| CL8932.Contig2_All | 63.68 | 63.7 | 72.2 | 48 | PKM | 0.275 | 0.000 | 0.817 | 0.016 | 0.291 | 0.000 |
| Unigene3383_All | 9.71 | 12.22 | 20.8 | 8 | PLAA | 5.754 | 0.021 | 4.529 | 0.028 | 4.286 | 0.031 |
| Unigene1017_All | 22.83 | 23 | 45.1 | 22 | PLIN3 | 0.752 | 0.511 | 0.328 | 0.020 | 0.492 | 0.060 |
| Unigene985_All | 10.3 | 10.36 | 35.3 | 5 | PPA1 | 3.532 | 0.055 | 1.213 | 0.810 | 0.160 | 0.027 |
| CL1333.Contig2_All | 16.06 | 16.07 | 46.3 | 9 | PPP2CB | 1.542 | 0.590 | 0.187 | 0.018 | 0.380 | 0.021 |
| Unigene840_All | 16.24 | 16.3 | 36 | 15 | PRDX3 | 2.070 | 0.261 | 7.586 | 0.018 | 4.699 | 0.384 |
| Unigene619_All | 20.02 | 20.02 | 31.9 | 12 | PRMT1 | 0.082 | 0.000 | 0.256 | 0.008 | 0.105 | 0.001 |
| CL4749.Contig1_All | 5.86 | 6.97 | 25.4 | 4 | PRPSAP1 | 2.312 | 0.044 | 6.546 | 0.040 | 1.871 | 0.045 |
| Unigene1136_All | 10.49 | 10.63 | 41.8 | 10 | PSMB1 | 1.500 | 0.427 | 3.981 | 0.007 | 1.690 | 0.872 |
| Unigene1850_All | 11.2 | 11.29 | 39 | 7 | PSMB3 | 0.711 | 0.675 | 3.873 | 0.048 | 0.637 | 0.692 |
| Unigene3005_All | 6.02 | 6.02 | 22.6 | 4 | PSMD14 | 1.644 | 0.044 | 7.943 | 0.035 | 0.738 | 0.058 |
| Unigene3612_All | 4.39 | 4.53 | 23.9 | 4 | PSMD8 | 2.443 | 0.133 | 0.177 | 0.024 | 1.306 | 0.767 |
| CL2142.Contig1_All | 26.15 | 26.24 | 55.3 | 19 | PTER | 2.911 | 0.038 | 3.598 | 0.006 | 1.038 | 0.760 |
| CL7668.Contig4_All | 114.26 | 114.73 | 69.8 | 87 | PYGL | 1.406 | 0.242 | 2.679 | 0.000 | 0.575 | 0.012 |
| Unigene1451_All | 21.25 | 21.27 | 69.6 | 15 | RAB7 | 0.817 | 0.406 | 0.209 | 0.032 | 1.009 | 0.848 |
| Unigene1783_All | 11.69 | 11.82 | 29.6 | 7 | RAD23B | 3.342 | 0.010 | 3.698 | 0.010 | 2.377 | 0.014 |
| Unigene6198_All | 4 | 4 | 8.7 | 2 | RALB | 3.733 | 0.042 | 1.318 | 0.070 | 5.012 | 0.040 |
| Unigene702_All | 15.55 | 15.68 | 52.3 | 15 | RAN | 0.718 | 0.469 | 0.171 | 0.003 | 1.282 | 0.448 |
| Unigene2249_All | 24.69 | 25.02 | 34.4 | 12 | RARS | 0.370 | 0.023 | 0.200 | 0.065 | 0.288 | 0.026 |
| Unigene3353_All | 15.96 | 16.14 | 64.9 | 13 | RBP7 | 1.117 | 0.778 | 0.302 | 0.004 | 0.887 | 0.152 |
| CL142.Contig2_All | 6.33 | 6.56 | 21.2 | 4 | RNASET2 | 1.995 | 0.406 | 2.535 | 0.049 | 3.373 | 0.082 |
| Unigene481_All | 16.47 | 16.82 | 45.3 | 8 | RPL13A | 0.619 | 0.093 | 0.233 | 0.013 | 0.711 | 0.242 |
| Unigene511_All | 11.03 | 11.1 | 37.6 | 9 | RPL24 | 0.256 | 0.031 | 0.203 | 0.008 | 0.391 | 0.201 |
| CL5814.Contig2_All | 7.07 | 7.37 | 26.8 | 8 | RPL3 | 0.219 | 0.058 | 0.240 | 0.027 | 0.299 | 0.074 |
| Unigene488_All | 12.11 | 12.2 | 30.8 | 9 | RPL6 | 0.479 | 0.281 | 0.041 | 0.002 | 0.445 | 0.012 |
| CL8134.Contig2_All | 7.68 | 7.77 | 30.9 | 4 | RPS18 | 0.046 | 0.007 | 0.470 | 0.032 | 1.247 | 0.456 |
| Unigene539_All | 22.23 | 22.36 | 59.1 | 12 | RPS2 | 0.161 | 0.021 | 0.150 | 0.026 | 0.437 | 0.010 |
| Unigene545_All | 16.39 | 16.49 | 64.1 | 12 | RPS27A | 0.560 | 0.347 | 4.571 | 0.002 | 2.312 | 0.006 |
| Unigene458_All | 27.43 | 27.48 | 57 | 20 | RPS3 | 0.470 | 0.172 | 0.273 | 0.037 | 0.766 | 0.287 |
| CL6507.Contig2_All | 34.77 | 34.83 | 24 | 21 | RRBP1 | 0.847 | 0.726 | 0.129 | 0.004 | 0.625 | 0.218 |
| Unigene2428_All | 15.56 | 15.71 | 30.7 | 10 | RTCB | 0.581 | 0.665 | 0.142 | 0.022 | 1.117 | 0.813 |
| CL3515.Contig3_All | 10.78 | 10.88 | 43.4 | 9 | S100A13 | 2.884 | 0.159 | 0.787 | 0.597 | 9.462 | 0.016 |
| Unigene27346_All | 60.46 | 60.52 | 56.9 | 37 | SARDH | 1.380 | 0.297 | 1.675 | 0.022 | 1.660 | 0.172 |
| Unigene1769_All | 20.3 | 20.4 | 31.9 | 11 | SARS | 0.824 | 0.871 | 0.084 | 0.027 | 0.515 | 0.252 |
| Unigene2187_All | 23.53 | 23.6 | 31.7 | 13 | SCP2 | 0.773 | 0.506 | 0.205 | 0.001 | 0.904 | 0.937 |
| CL4057.Contig2_All | 18.6 | 18.66 | 59.7 | 11 | SDR16C5 | 4.286 | 0.162 | 9.205 | 0.031 | 12.823 | 0.006 |
| CL4112.Contig2_All | 30.78 | 30.86 | 63 | 22 | SEC14L2 | 2.291 | 0.358 | 4.920 | 0.035 | 4.699 | 0.068 |
| Unigene734_All | 63.69 | 63.72 | 89 | 95 | SELENBP1 | 1.380 | 0.093 | 3.945 | 0.000 | 0.530 | 0.046 |
| Unigene9050_All | 26.45 | 27.69 | 50.9 | 26 | SERPINA1 | 0.530 | 0.226 | 1.585 | 0.832 | 0.535 | 0.033 |
| Unigene69728_All | 42.33 | 42.54 | 65.2 | 38 | SERPINB10 | 0.437 | 0.046 | 0.773 | 0.585 | 1.038 | 0.849 |
| CL1613.Contig1_All | 27.93 | 28.05 | 45.2 | 15 | SERPINC1 | 0.406 | 0.473 | 2.559 | 0.031 | 2.312 | 0.047 |
| Unigene537_All | 13.92 | 13.99 | 34.2 | 9 | SET | 0.322 | 0.010 | 1.213 | 0.409 | 2.070 | 0.504 |
| Unigene2046_All | 30.59 | 30.64 | 52.3 | 20 | SHMT1 | 0.437 | 0.320 | 0.207 | 0.025 | 0.256 | 0.020 |
| Unigene30041_All | 3.23 | 3.42 | 7.5 | 3 | SLC16A1 | 0.711 | 0.229 | 0.555 | 0.019 | 0.614 | 0.045 |
| Unigene4530_All | 4.43 | 4.6 | 13.9 | 3 | SLC25A1 | 0.175 | 0.037 | 1.445 | 0.893 | 0.887 | 0.454 |
| CL1967.Contig2_All | 41.52 | 42.19 | 52.4 | 33 | SLC25A13 | 2.992 | 0.282 | 3.404 | 0.297 | 5.200 | 0.017 |
| Unigene3785_All | 12.6 | 12.65 | 29.1 | 7 | SLC25A24 | 0.895 | 0.694 | 1.393 | 0.060 | 1.660 | 0.027 |
| CL600.Contig3_All | 22.84 | 23.09 | 59.1 | 16 | SLC25A5 | 0.449 | 0.011 | 0.263 | 0.033 | 0.625 | 0.138 |
| Unigene18341_All | 1.85 | 2.09 | 4.9 | 2 | SLC2A9 | 11.588 | 0.036 | 19.588 | 0.032 | 8.395 | 0.037 |
| Unigene946_All | 46.01 | 46.13 | 47.5 | 31 | SND1 | 0.711 | 0.431 | 0.433 | 0.020 | 0.667 | 0.441 |
| Unigene4549_All | 2.25 | 2.4 | 8 | 2 | SNRPA | 0.017 | 0.046 | 0.863 | 0.777 | 1.000 | 0.986 |
| Unigene8249_All | 12.36 | 13.13 | 27.1 | 7 | SNX5 | 1.097 | 0.817 | 2.051 | 0.713 | 3.908 | 0.047 |
| CL8066.Contig2_All | 10.07 | 10.08 | 61.9 | 14 | SOD1 | 1.393 | 0.003 | 3.698 | 0.004 | 1.660 | 0.061 |
| Unigene1344_All | 11.24 | 11.31 | 41.2 | 10 | SOD2 | 0.661 | 0.893 | 1.690 | 0.297 | 2.443 | 0.030 |
| CL2043.Contig1_All | 18.51 | 18.7 | 19.4 | 13 | SPEN | 0.802 | 0.323 | 0.121 | 0.020 | 0.406 | 0.027 |
| CL4279.Contig3_All | 262.34 | 262.33 | 62.4 | 164 | SPTAN1 | 1.459 | 0.066 | 2.965 | 0.000 | 2.911 | 0.000 |
| Unigene4675_All | 20 | 20.08 | 37.2 | 11 | SRM | 9.205 | 0.095 | 3.162 | 0.944 | 10.186 | 0.033 |
| Unigene798_All | 14.01 | 16.4 | 41.4 | 13 | SRSF9 | 0.809 | 0.474 | 0.120 | 0.000 | 0.718 | 0.178 |
| Unigene2598_All | 34.58 | 34.59 | 29.6 | 19 | STAT1 | 0.157 | 0.002 | 0.099 | 0.002 | 0.231 | 0.000 |
| Unigene1217_All | 19.97 | 20.01 | 51.9 | 19 | STOM | 0.625 | 0.127 | 0.258 | 0.001 | 0.511 | 0.063 |
| Unigene2767_All | 10.55 | 10.68 | 33.4 | 7 | STOML2 | 1.406 | 0.907 | 4.093 | 0.034 | 3.767 | 0.135 |
| Unigene2399_All | 17.57 | 17.68 | 18.8 | 9 | SUGP2 | 0.759 | 0.721 | 0.409 | 0.006 | 0.787 | 0.590 |
| CL5810.Contig2_All | 17.67 | 18.16 | 76.7 | 14 | SULT3A1 | 2.606 | 0.040 | 0.530 | 0.528 | 1.738 | 0.306 |
| CL3099.Contig2_All | 19.16 | 19.34 | 38.4 | 16 | SULT6B1 | 0.879 | 0.433 | 0.483 | 0.040 | 0.570 | 0.095 |
| CL7427.Contig2_All | 8.87 | 9.14 | 69.7 | 8 | TAF1C | 0.614 | 0.097 | 2.655 | 0.048 | 1.159 | 0.166 |
| Unigene9946_All | 43.04 | 43.07 | 58.8 | 31 | TAF4 | 1.086 | 0.866 | 0.614 | 0.652 | 2.443 | 0.009 |
| Unigene941_All | 43.94 | 44.09 | 72.1 | 35 | TALDO1 | 0.479 | 0.012 | 1.770 | 0.001 | 0.667 | 0.314 |
| Unigene787_All | 26.53 | 26.66 | 37.3 | 18 | TCP1 | 0.380 | 0.008 | 0.151 | 0.002 | 0.879 | 0.688 |
| Unigene950_All | 103.4 | 103.45 | 84 | 167 | TF | 0.520 | 0.003 | 2.014 | 0.024 | 0.871 | 0.040 |
| CL3702.Contig1_All | 4 | 4.02 | 7.9 | 3 | THA1 | 5.970 | 0.042 | 15.849 | 0.034 | 5.861 | 0.040 |
| Unigene857_All | 61.66 | 62.41 | 73.4 | 56 | TKT | 1.057 | 0.386 | 0.069 | 0.000 | 0.840 | 0.022 |
| CL8754.Contig2_All | 146.39 | 146.33 | 46.6 | 95 | TLN1 | 0.904 | 0.078 | 0.575 | 0.016 | 1.941 | 0.462 |
| CL8810.Contig2_All | 12.78 | 13.99 | 9.6 | 9 | TOP2B | 0.273 | 0.019 | 0.244 | 0.098 | 0.667 | 0.468 |
| CL8857.Contig2_All | 37.01 | 37.03 | 75.4 | 68 | TPI1 | 1.000 | 0.589 | 4.286 | 0.000 | 0.938 | 0.147 |
| Unigene13577_All | 34.28 | 35.3 | 60.2 | 20 | TPM2 | 0.752 | 0.096 | 0.637 | 0.004 | 0.780 | 0.608 |
| Unigene529_All | 22.35 | 22.49 | 74 | 18 | TPT1 | 0.619 | 0.002 | 0.871 | 0.290 | 0.817 | 0.024 |
| Unigene9728_All | 3.6 | 3.72 | 13.3 | 2 | TRAPPC5 | 87.902 | 0.017 | 62.517 | 0.019 | 86.298 | 0.018 |
| CL6519.Contig3_All | 38.79 | 38.92 | 43.6 | 22 | TRIM39 | 1.117 | 0.741 | 0.149 | 0.004 | 0.387 | 0.074 |
| Unigene564_All | 27.11 | 27.15 | 42.9 | 25 | TUBB4B | 1.009 | 0.138 | 0.313 | 0.039 | 0.840 | 0.252 |
| Unigene1944_All | 14.32 | 14.34 | 79.7 | 10 | TXNDC17 | 1.556 | 0.843 | 5.395 | 0.028 | 2.032 | 0.711 |
| Unigene1238_All | 36.04 | 37.36 | 60.3 | 30 | TXNDC5 | 0.233 | 0.007 | 0.824 | 0.090 | 0.520 | 0.037 |
| Unigene1607_All | 12.2 | 12.34 | 26.8 | 7 | TXNRD3 | 0.227 | 0.291 | 0.366 | 0.089 | 0.145 | 0.041 |
| Unigene3260_All | 36.9 | 37.02 | 64.6 | 22 | UGDH | 2.188 | 0.007 | 0.973 | 0.662 | 0.395 | 0.185 |
| Unigene4797_All | 7.89 | 8.18 | 15.8 | 6 | UGT3A1 | 1.706 | 0.043 | 1.854 | 0.046 | 1.282 | 0.098 |
| Unigene3307_All | 40.57 | 40.62 | 66.7 | 47 | UPB1 | 0.904 | 0.485 | 2.249 | 0.008 | 0.520 | 0.078 |
| Unigene1290_All | 23.93 | 23.99 | 47.8 | 15 | UQCRC2 | 3.048 | 0.034 | 0.802 | 0.767 | 2.780 | 0.475 |
| Unigene1480_All | 10.73 | 10.78 | 17.9 | 6 | USP14 | 3.162 | 0.046 | 0.787 | 0.706 | 1.941 | 0.219 |
| CL2425.Contig1_All | 6.13 | 6.16 | 6.1 | 3 | UTRN | 0.194 | 0.043 | 0.840 | 0.669 | 0.172 | 0.024 |
| Unigene4599_All | 23.7 | 23.73 | 45.4 | 16 | VAT1 | 2.780 | 0.066 | 4.286 | 0.007 | 2.032 | 0.075 |
| Unigene876_All | 61.87 | 61.87 | 64.7 | 43 | VCP | 0.297 | 0.015 | 0.291 | 0.006 | 0.511 | 0.021 |
| Unigene623_All | 34.11 | 34.14 | 70.3 | 47 | VDAC2 | 0.625 | 0.285 | 2.168 | 0.027 | 0.929 | 0.335 |
| Unigene528_All | 54.52 | 57.57 | 61.9 | 106 | VIM4 | 0.973 | 0.949 | 0.258 | 0.001 | 2.014 | 0.001 |
| Unigene41122_All | 23.67 | 23.73 | 51.5 | 20 | VNN1 | 0.497 | 0.915 | 2.630 | 0.001 | 1.368 | 0.174 |
| CL7734.Contig1_All | 63.89 | 63.91 | 60 | 52 | VPS54 | 1.706 | 0.000 | 1.047 | 0.839 | 0.824 | 0.126 |
| CL3158.Contig3_All | 31.7 | 31.92 | 22.6 | 23 | VTGA2 | 0.278 | 0.174 | 0.506 | 0.347 | 0.100 | 0.043 |
| Unigene4436_All | 16.04 | 16.07 | 20.6 | 8 | XRCC6 | 1.644 | 0.003 | 0.692 | 0.021 | 1.294 | 0.003 |
| Unigene1132_All | 27.13 | 27.21 | 21.7 | 16 | XRN2 | 0.281 | 0.024 | 0.501 | 0.081 | 1.459 | 0.982 |
| Unigene657_All | 40.07 | 40.11 | 75.3 | 27 | YWHAE | 0.525 | 0.380 | 0.294 | 0.006 | 0.920 | 0.110 |

Orange and green colors denote a higher and lower expression level than the control.
